# Feelings of Knowing - Fundamental Interoceptive Patterns (FoK-FIP): a magnetic monopole-like “pure mental” process fundamental to subjective feelings and self-awareness

**DOI:** 10.1080/19420889.2021.2023280

**Published:** 2022-02-16

**Authors:** Holly Pollard-Wright

**Affiliations:** Institute of Electrical and Electronics Engineers (IEEE), The National Coalition of Independent Scholars (NCIS)

**Keywords:** Magnetic monopoles, observing ego, interoceptive cognition, Feelings of Knowing, consciousness

## Abstract

The Feelings of Knowing - Fundamental Interoceptive Patterns (FoK-FIP) is a transdisciplinary theory developed to explain elusive phenomena suspected to exist that do not easily lend themselves to empirical measurement. The FoK-FIP theory posits that specialized self-generated biomagnetism and “pure mental” process share similarities with the hypothetical elementary particle described in particle physics, magnetic monopoles with a magnetic charge. Feelings of Knowing (FoK) are “awareness charge” that are self-generated events. Fundamental Interoceptive Patterns (FIP) are restricted oscillatory magnetic fields that are FoK caused phenomena. Further, FoK produces “cognitive force,” an observing ego representing specialized interoceptive awareness. Through embodied states, FoK-FIP acts as a “biological node,” an informational processing unit in which physiological signals and an observing ego’s sensations or feelings are centered. An observing ego cognitively broadcasts using specialized small magnetic signals and four phases of a narrowed range of interoceptive signals. By defining interoceptive signals (i.e., signals of the body’s internal state) using FoK-FIP through cognitive broadcasting, an observing ego creates a world it projects around itself. This process is understood through the components map with interoceptive markers (IMs), a novel algorithm based on biological evolution. FoK-FIP-related predictions are described as are empirical studies to test aspects of the theory. The FoK-FIP theory details a path to wellbeing based on a sense of control and capacity for self-care. Mental stability is thought to change as a function of an observing ego’s volitional reactions.

## Introduction

This FoK-FIP theory repurposes two terms: FoK, which has been in use for at least 25 years and the term observing ego, a psychoanalytical construct introduced by the Austrian neurologist and founder of psychoanalysis Sigmund Freud. The FoK-FIP theory is congruent with philosophical and existentialist works that have addressed how the body influences conscious experience and is congruent with existing scholarship of contemporary thinkers (e.g., Varela et al., *The Embodied Mind: Cognitive Science and Human Experience*). In the FoK-FIP theory, interoceptive cognition is understood as one of three types of consciousness that are different manifestations of the same phenomenon. It posits that the mind is a fundamental entity without beginning or end, and one with infinite potential that is not entirely conceivable. Nevertheless, through this existing infinite potential, the mind causes change to occur. Further, our Universe of approximately 68% dark energy, 27% dark matter, and 5% normal matter represents potential energies or change. These are potential energies because of their relationship to the mind. Understood is that the mind can be partly explained by an abstract idea the law of conservation of energy (the mind cannot be created or destroyed) [1], but potential energies convert or transform from one form into another. Though the mind does not change when it manifests its infinite potential, as a consequence, change occurs and causal dependence emerges. As such, change represents aspects of the mind in which one event, process, state, or object all relate to this fundamental entity. When potential energies convert or transform from one form into another, dynamic differences occur through interdependent relationships. Through the mind that caused change, consciousness emerged, followed by electromagnetic radiation consciousness in which interoceptive cognition consciousness follows. Interoceptive cognition consciousness exists through interdependent embodied relationships, a concept congruent with electromagnetic field theories (or EM field theories). Importantly, FoK-FIP are part of the dynamics of embodied relationships from which interoceptive cognition consciousness emerges. In this FoK-FIP theory, interoceptive cognition consciousness (i.e., “different manifestation of the same phenomenon”) is congruent with Maxwell’s equations, a classical theory of electromagnetic radiation that describes electricity, magnetism, and light as different manifestations of the same phenomenon. James Clerk Maxwell, a Scottish mathematician and scientist, described the unification of electric and magnetic field theory into classical electromagnetism. Further, FoK-FIP, specialized self-generated biomagnetism and “pure mental” process, is congruent with a particle physics quantum electrodynamic counterpart, magnetic monopoles. As such, FoK-FIP, are a monopole-like process that give rise to a unique property, an observing ego congruent with magnetic monopoles, an elusive particle of quantum physics with unique properties. Observing egos are elusive emergent properties not seen but subjectively suspected to exist since the beginning of interoceptive cognition consciousness. When aspects of the mind are united through embodied states with the same substrate, cooperative conditions occur in which observing egos represent logical outcomes.

An observing ego and invisible viewpoint of the physical world helps explain conscious events and why subjective reality works the way it does. Similarly, magnetic monopoles have never been observed; instead, magnets exist only in the form of dipoles with a north and south pole. Accordingly, the magnetism observed daily in the physical world can be attributed to the movement of electric charges entirely consistent with Maxwell’s equations. Nevertheless, just because classical electromagnetic theories are consistent with our observation does not mean that there are no magnetic monopoles, only that there are no magnetic monopoles anywhere that have been observed, an argument with which Nobel Laureate Pierre Curie agreed [2]. Curie discussed the possibility of the magnetic monopole and could find no reason to discount their existence. Later Nobel Laureate Paul Dirac showed that when Maxwell’s equations are extended to include a magnetic monopole, electric charge can exist only in discrete values through a “quantization” process [2]. Electric charge is a requirement of quantum mechanics, and Dirac’s work showed that classical electromagnetism and quantum electrodynamics were compatible theories in this sense. The FoK-FIP theory is congruent with Dirac’s work, in which duality is the physical sensed by an observing ego interconnected with interoceptive signals (i.e., signals of the body’s internal state) through a biological node (i.e., FoK-FIP) that centers its sensations or feelings. An observing ego is congruent with the emergence of awareness, or sentience. Due to FoK-FIP, an observing ego can sense specialized small magnetic signals and experiences them through four phases of a narrowed range of interoceptive signals. These signals act as stimuli that an observing ego uses to create its “cognitive position” to evaluate signals. By doing this via perception, an observing ego can cognitively differentiate signals. As such, the integration of interoceptive signals (i.e., signals of the body’s internal state) by an observing ego is cognitive broadcasting, the subjective processing of signals. Further, an observing ego represents discrete “cognitive value,” a value that would be obtained by subjective measurement congruent with discrete values of a “quantization” process. Over the years, a unified universe concept has been evolving through scientific research that include Sir Isaac Newton’s laws of motion as three laws that explain the gravitational force of classical mechanics. In the nineteenth century, as previously mentioned, scientist James Clerk Maxwell unified optics with electromagnetism by showing that electricity and magnetism are two different aspects of the same phenomena and electromagnetic waves were light waves. In the twentieth century, theoretical physicist Albert Einstein united time and space, and it was concluded from a famous thought experiment (i.e., Einstein–Podolsky–Rosen Paradox or EPR experiment) that subatomic particles are interdependent. Both light and matter have a dual nature where particles (i.e., photons and electrons and other particles of matter) sometimes appear as particles and sometimes appear as waves. Whether a particle is in the wave or particle state depends on the role of the observer. If an individual tries to observe the particle in its wave state, it becomes a particle, and unobserved remains in the wave state [3]. This FoK-FIP theory builds on the EPR experiment and improves upon that theoretical construct by defining the observer through a transdisciplinary approach. The FoK-FIP theory is congruent with particle physics theories and a broad range of other theories, from, for example, neurobiology, biological psychiatry or biopsychiatry, psychology, and bioinspired computing.

This article includes a theory of wellbeing based on a sense of control and capacity for self-care. The FoK-FIP theory places mental stability as the central construct that gives rise to a sense of wellbeing through an observing ego. Stability is defined through the FoK-FIP that an observing ego uses during cognitive broadcasting to differentiate interoceptive signals. An observing ego has relatively little access to interoceptive signals but acts to intervene in response to emerging signals as either an “impulsive reaction” or a “reaction with forethought.” Mental stability is thought to change as a function of an observing ego’s volitional reactions. As such, an increase or decrease in stability occurs through goal-directed reactions. Success or failure is defined by an observing ego’s ability to anchor its reactivity to FoK-FIP ultimately. An observing ego can become attached or aversive to pleasant or unpleasant FoK-FIP. The idea that all conscious percepts are associated with either attachment or aversion is well-trodden ground, especially in Buddhist psychology [4,5], as is the distinction of reacting versus responding (see Maex, 2011). The FoK-FIP theory defines attachment or aversion in relation to FoK-FIP with quantum mechanics implications. It improves upon reaction-related conscious percept models in ways that open avenues for novel empirical research. Research ideas discussed in this article include the components map model with interoceptive markers (IMs), a novel algorithm based on biological evolution that may help develop new and robust competing techniques. For example, through the single-celled organism model, FoK-FIP is understood as DNA encoded intracellular self-generated specialized biomagnetism. Specialized interoceptive awareness (i.e., an observing ego) emerges from FoK. FoK-FIP is congruent with new methodologies from engineering solutions to complex computing problems. As such, computing problems with the slime mold *Physarum polycephalum* model. This single-cell visible by the unaided eye [6] exhibits self-awareness and self-expressiveness while adapting to changes in its environment [7]. Additionally, this article includes proposed research and predictions with global implications for mental health and ecology fields. These research ideas are not based on simple observations and research studies in a single center. Instead, in some instances, they are based on reports from clinical trials and cohort studies. Scientific hypotheses are essential for progress in rapidly developing academic disciplines. Hypotheses with unconventional ideas may open gates to critics and conservative remarks [8]. The research ideas proposed in this article call for multicenter and interdisciplinary research with the intent of potentially launching influential scientific directions. A process through which professional debates generate realistic ideas that are the fuel through which scientific discovery “marches on.”

### FoK-FIP in the physical world

The physical world is posited to be a shared reality not as stable as it appears, a concept congruent with theoretical physics and basic Buddhist concepts. Further, through FoK-FIP in the physical sense, this reality occurs when two different processing activities, subjective and objective, are melded. Understood is that the perceptions of an observing ego represent the subjective mind and interoceptive signals (i.e., signals of the body’s internal state) the objective mind. The objective and subjective minds represent different interoceptive experiences of equal reality through embodied states. The related activities of these systems connect through an informational processing unit (also biological node), FoK-FIP. Through specialized self-generated biomagnetism (i.e., FoK-FIP), physiological signals along with sensations or feelings get centered:

1. The subjective mind – an observing ego and the emergent conscious event is the viewpoint that defines the objective mind or interoceptive signals (i.e., signals of the body’s internal state). Through reactions, cognitive broadcasting occurs as a function of subjective informational processing activities. The FoK of the FoK-FIP theory gives rise to this specialized interoceptive awareness. The FIP (i.e., restricted oscillatory magnetic fields) rise to a sense of specialized small magnetic signals that an observing ego experiences through four phases of a specific type of interoceptive sensation (also narrowed range of interoceptive signals). FoK-FIP centers an observing ego’s sensations or feelings to sense and differentiate interoceptive signals.
2. The objective mind – the body’s internal state, interoceptive signals, and the emergent unconscious (also preconscious or subconscious) event. Through nonconscious processes, signals occur as a function of objective informational processing activities in which physiological signals are centered through FoK-FIP.

**Figure 1.**
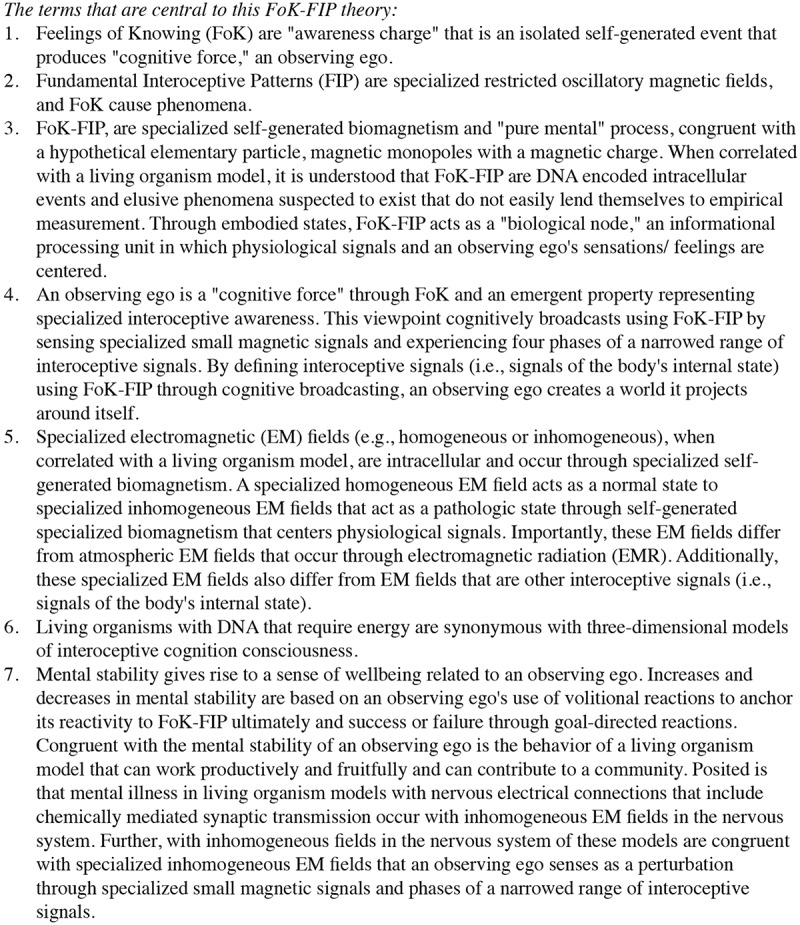
The terms that are central to this FoK-FIP theory.

*The terms that are central to this FoK-FIP theory* [Figure 1]:

Feedback loops are the mechanism by which FoK-FIP exert an effect. This process includes a bidirectional relationship between FoK-FIP and the subjective and objective minds. Through embodied states, FoK-FIP centers physiological signals and the sensations or feelings of an observing ego in which a close relationship to a living organism model gets created through higher-end cognitive broadcasting (to varying degrees). Part of the cognitive broadcasting process when the model is human includes the condition of being informative (also “informativeness”) [9]. This FoK-FIP theory builds upon other models of consciousness and improves other models of interoception by melding them with computer science models such as those created by Jay Wright Forrester, an American computer engineer and systems scientist. Forrester is one of the inventors of magnetic core memory, the predominant form of random access computer memory congruent with this theory where FoK-FIP is a magnetic monopole-like “pure mental” process. In the process of broadcasting for higher-end cognition, the subjective mind (i.e., an observing ego) understands its relationship to the objective mind (i.e., interoceptive signals) and FoK-FIP due to the behavior of a particular human model. As such, higher-end cognition content supports knowledge of this interdependent relationship through conceptual mental content. Higher-end “tangible” content creates an observing ego’s belief in a solid reality to what it is perceiving that includes three-dimensional imagery (e.g., other living organisms, places, and things) in a world that functions through feedback loops (e.g., physical, electrical, social, political, biological, and medical systems in the body) [10]. An observing ego is posited to create a world through cognitively broadcasting that it projects around itself, with a continuing cast of characters whose close relationship with a particular human model plays a starring role. Further, because the physical world is a shared reality, it often includes other characters of markedly different types thrown together by circumstances. FoK-FIP creates the “sensory theme” of higher-end cognition by centering sensations or feelings that often include attachment or aversion while observing egos struggle to understand their relationship to a particular human model. Through an embodied state, life plays out in which higher-end cognition may include situations (e.g., population, resources, capital investment and industry, investment and agriculture, and the accumulated pollution in the world) that are all networks fundamentally of feedback loops [10].

Specialized interoceptive awareness (i.e., an observing ego) includes varying sensitivity to specialized small magnetic signals and four phases of a narrowed range of interoceptive signals. The volitional reactions of an observing ego act as feedback to the biological node that causes FoK-FIP to change the way it centers signals, or signal centering remains ‘status quo.’ An observing ego’s volitional reactions promote or prevent cognitive actions [11]. An observing ego’s sensitivity to FoK-FIP ultimately is how the signal centering through the biological node “bends back on” to affect an overall self-reflective cognitive process. Impulsive reactions alone and impulsive reactions followed by reactions with forethought are part of this process. These volitional reactions act as excitatory or inhibitory responses of an observing ego with varying degrees of FoK-FIP sensitivity. When FoK-FIP are understood as DNA encoded, then intracellular events that include the emergence of specialized interoceptive awareness act as conditions for transmitting information from one evolutionary stage to the next. Through this interdependent relationship, specialized self-generated biomagnetism FoK-FIP represents:

1. A stable mechanism for encoding, transmitting, and decoding signals.
2. The possibility of alteration in the code taking place.
3. A mechanism for selecting only those signals for transmission that are favorable for survival.

Living organisms with DNA that require energy are posited to be the models of the embodied system (i.e., the relationship between the subjective mind and the objective mind connected through a biological node). Single-celled organisms, are the models representing how this system began. Through single-celled organisms FoK-FIP are DNA encoded intracellular events in which a specialized biomagnetism phenomenon synthesizes subjective and objective informational processing activities. This functional process enables feedback loops to produce end products, in which signaling ultimately affects a model’s phenotype as the final effect. Further, single-celled organism models evolved so that two model types emerged to represent the evolution of this relationship in which electrically mediated junctional transmission may be older than chemically mediated synaptic transmission. Importantly, observing egos derive varying degrees of a concept of self through a close relationship with a model of interoceptive cognition consciousness:

1. Living organisms (e.g., plants and slime mold) with non-nervous electrical connections that include electrically mediated junctional transmission. The FoK-FIP theory posits that these models imply that interoceptive cognition consciousness does not include emotions and moods. Further, congruent with these models is that an observing ego does not suffer emotional pain while cognitively broadcasting. These models imply basic behavior includes reacting to electrical potentials that motivate reactions devoid of emotional/mood response.
2. Living organisms (e.g., humans and other animals) with nervous electrical connections that include chemically mediated synaptic transmission. The FoK-FIP theory posits that these models imply interoceptive cognition consciousness does include emotions/moods (to varying degrees). In a broad sense, suffering or pain may be an experience of unpleasantness and aversion to a specific type of interoceptive sensation in a body region associated with the perception of harm or threat. Importantly, FoK-FIP is congruent with a specific type of interoceptive sensation in a body region of a living organism model rather than an organ. This specific interoceptive sensation differs from hunger, thirst, respiratory and cardiac signals, which are all interoceptive signals [12]. Further, congruent with these models is that an observing ego suffers and feels pain while cognitively broadcasting (to varying degrees) where mental stability is a function of goal-directed volitional reactions to procure or avoid FoK-FIP ultimately.

This FoK-FIP theory builds upon contemporary theories [13,14] that include the predictive coding process (i.e., a neuroscience model).The objective mind or interoceptive signals (i.e., signals of the body’s internal state) constantly generates and updates information while connected to the biological node. Objective signaling events that occur represent bottom-up encoded input expectations. The subjective mind or observing ego constantly defines signals as conscious events congruent with the objective mind’s activities. Feedback loops are the mechanisms where both the subjective and objective minds connect to FoK-FIP. The feedback loops between an observing ego and biological node provide the stimulus-response coordination of the system that creates top-down interoceptive predictions. Further, when an observing ego cognitively broadcasts, FoK-FIP center its sensations or feelings to interoceptive signals, creating strong but vague beliefs that something will happen. This FoK-FIP theory builds upon the work of Jerome Seymour Bruner, a psychologist who made significant contributions to cognitive psychology and learning, including how needs, motivations, and expectations influence perception. This FoK-FIP theory defines perception as a cognitive broadcasting subjective process. Through an observing ego, intellectual development is a continuous process of defining signals. Due to specialized interoceptive awareness, perception synthesizes the interaction between sensory stimuli (bottom-up) and conceptual knowledge (top-down). In the cognitive broadcasting process, when an observing ego defines signals, it fills in visual information to make sense of bottom-up input that includes the visual field. As such, an observing ego ultimately perceives and involuntarily experiences visual information such as depth, people, place, and things. An observing ego can cognitively broadcast to create a world that it projects around itself by doing this.

This FoK-FIP theory posits that the laws of quantum mechanics determine the ultimate empirical measurement of mental stability. Further, these laws are most noticeable at the atomic level and become relevant through an observing ego that broadcasts using FoK-FIP, including higher-end cognition. Metacognitive awareness is a cognitive broadcasting process. The FoK-FIP theory posits that it builds on work that accelerates progress in understanding the role of interoception in mental health organized around three themes: interoceptive assessment, interoceptive integration, and interoceptive psychopathology [15]. When FoK-FIP are understood as isolated self-generated DNA encoded intracellular events, specialized EM fields (e.g., homogeneous or inhomogeneous) occur. Understood through living organism models is that dysfunction of interoception, the sense of the body’s internal state that can be both conscious and non-conscious, is increasingly recognized as an important component of different mental illnesses such as anxiety disorders, mood disorders, eating disorders, addictive disorders, and somatic symptom disorders [15]. The models’ disorders are posited to occur with inhomogeneous EM fields in the nervous system. Further, congruent with inhomogeneous EM fields in the nervous system of models is the specialized inhomogeneous EM fields that observing egos can sense as a perturbation (i.e., through specialized small magnetic signals and phases of a narrowed range of interoceptive signals). Interoceptive signaling has been considered a component process of reflexes, urges, feelings, drives, adaptive responses, and cognitive and emotional experiences, highlighting its contributions to maintaining homeostatic functioning, body regulation, and survival [15]. This FoK-FIP theory improves upon other interoception models by defining the relationship between an observing ego and FoK-FIP and an observing ego’s close relationship to a model of interoceptive cognition consciousness. Additionally, the moment an observing ego through cognitive broadcasting identifies with FoK-FIP and pursues them, its suffering follows due to stress reactivity and response. In this cognitive process, mental instability ensues by which an observing ego does not decide on the particular course of action it commits. Instead, volitional processes are automized by an observing ego unable to anticipate FoK-FIP. An oberving ego does not use a goal-orientated reaction to anchor its reactivity to FoK-FIP ultimately. In contrast, building upon mindfulness in modern medicine and healthcare (see Maex, 2011), when an observing ego can anticipate FoK-FIP and decides to commit to a particular volition reaction as a goal-directed course of action, this process changes its relationship to FoK-FIP. An observing ego using goal-directed reactions, particularly a volitional reaction sequence of an impulsive reaction followed by reaction with forethought, can change its automized habits over time. The outcome of this process is an observing ego with volitional reactions that it uses to anchor its reactivity to FoK-FIP willfully, and mental stability follows because it cognitively broadcasts differently. As such, while defining signals, an observing ego can “see a thought as a thought, a feeling as a feeling,” which research has shown is a key element for success in mindfulness training [5]. Further, part of this success comes through an observing ego that does not judge and criticize itself for cognitively broadcasting in which it has relatively little access to this process that includes interoceptive signals that create thoughts and feelings. By understanding how the system functions, an observing ego empowers itself with knowledge of when to intercede to increase its sense of control and capacity for self-care.

This FoK-FIP theory builds on biological psychiatry that began more than 100 years ago and was predated by interest in linking body-brain interactions with conscious experience roughly 2,500 years ago [15]. Building upon interoception and mental health roadmap research (see Khalsa et al., 2018) [15] are FoK-FIP features related to an observing ego:

1. Attention: An observing ego observes internal body sensations in which small magnetic and a narrowed range of interoceptive signals center its sensations or feelings related to interoceptive signals (i.e., signals of the body’s internal state).
2. Detection: Presence or absence of conscious report through an observing ego. This concept is understood through the behavior of a human model in which verbal feedback occurs.
3. Magnitude: Perceived intensity by an observing ego of FoK-FIP. This concept is understood through the behavior of living organism models that when human verbal feedback may occur.
4. Discrimination: An observing ego localizes sensation using either small magnetic signals or the four phases of a narrowed range of interoceptive signals and differentiates it from other sensations. Discrimination is understood through living organism models to include a specific type of interoceptive sensation occurring in a specific body region.
5. Accuracy (Sensitivity): Correct and precise monitoring of an observing ego. A measure of the degree to which an observing ego can accurately perceive the internal embodied state that can vary across interoceptive domains [12].
6. Insight: Metacognitive evaluation of an observing ego’s experience/performance (e.g., confidence-accuracy correspondence). This concept is understood through the behavior of a human model that includes verbal feedback.
7. Sensibility: Self-perceived tendency of an observing ego to focus on small magnetic signals or four phases of a narrowed range of interoceptive signals that center its sensations or feelings to sense and integrate interoceptive signals. Interoceptive sensibility reflects the propensity of an observing ego to become aware of interoceptive information. This awareness focused internally can vary across interoceptive domains [12]. This sensibility can be understood through the behavior of living organism models, including verbal feedback that reveals a self-perceived tendency to focus on interoceptive stimuli (trait measure).
8. Self-report Scales: Psychometric assessment of an observing ego via state/trait measure. This scale is understood through the responses of a human model via a questionnaire.

### FoK-FIP and how the subjective mind (i.e., an observing ego) perceives the objective mind (i.e., interoceptive signals)

Understood through living organism models, FoK-FIP, specialized self-generated biomagnetism, that are DNA encoded intracellular events, cause the membrane potential of a specific cell location in a region of the body to rise and fall rapidly. This process represents specialized depolarization that causes the change in electrical potential associated with the passage of an impulse along the membrane of adjacent cells (e.g., nerve cells, or cells of plants). The FoK-FIP theory posits that specialized depolarization mechanism is congruent with a growing list of evidence that the cell membrane elements involved in the regulation of cell charge have more important roles in physiological and pathological processes than previously thought [16]. Further, building upon other theories through specialized depolarization, FoK-FIP influence processes such as cell motility and cell invasiveness that can trigger immune reactions and the facilitation of the recruitment of signaling molecules to the inner leaflet of the plasma membrane in cell receptors microclusters [16]. FoK, “awareness charge,” is posited to accumulate on opposite sides of cell membranes, and because of this, the potential for inducing varying degrees of an electric current exists. Further, through FoK-FIP, specialized intracellular self-generated and self-propagating transverse waves of oscillating magnetic-electric fields occur. The restricted magnetic fields, FIP that oscillate as a function of FoK, will produce an electric field, and an electric field that changes as a function of FoK will produce a magnetic field. These specialized EM fields (e.g., homogeneous or inhomogeneous), in turn, exert electric and magnetic forces on the cells. FoK-FIP acts as the biological node that typically links small magnetic signals to an accompanying electrical field where interactions occur between cells and cells or nerve fibers. During this process an observing ego emerges that begins to cognitively broadcast automatically. As such, FoK, through an observing ego, have an emergent property that makes the FoK-FIP act like small magnets sensitive to external magnetic fields. An observing ego acts as a “cognitive magnetometer” that can sense a weaker field than the Earth’s magnetic field. This specialized interoceptive process occurs through an embodied state where electromagnetic fields propagate that follow the laws of physics, in which no changing magnetic field can be isolated from an electric field, and where there is a magnetic field there is an electric current. Importantly through FoK-FIP, an observing ego experiences a narrowed range of interoceptive signals, and acting as a “cognitive magnetometer,” it senses specialized small magnetic signals.

An observing ego, the magnetic sensor, has varying interoceptive sensitivity, which makes its reactivity uncertain but sets the fundamental limit on the smallest external magnetic field that this specialized type of interoceptive awareness can sense. This concept is congruent with particle physics in which an elementary particle (i.e., various fundamental particles) is a subatomic particle, and ordinary matter is composed of atoms, the smallest unit that forms a chemical element. Atoms have the fundamental property of spin, making them act like small magnets that are sensitive to external magnetic fields. Accordingly, atoms can be used as magnetic sensors, but each atomic spin has a quantum uncertainty that sets the fundamental limit on the smallest external magnetic fields that the atoms can sense. The ultimate sensitivity of any measurement is posited to be determined by the laws of quantum mechanics and the laws of quantum mechanics congruent with an observing ego’s sensitivity to FoK-FIP. Through volitional reactions that act as feedback to the biological node, an observing ego causes FoK-FIP to change the way it centers sensations or feelings, or they remain “status quo.” In this process, an observing ego can suppress specialized self-generated biomagnetism-related errors of pure quantum mechanical origin congruent with electromagnetic (EM) energy sources of errors (e.g., through radio waves). Most magnetic activity related to FoK-FIP that centers physiological signals is posited to occur at extremely low frequencies. In this process, extremely low frequencies related to electrical activity occur congruently with an observing ego with varying degrees of FoK-FIP sensitivity. When understood through their relationship to living organism models, observing egos are specialized interoceptive awareness interconnected with the micro values of the electromagnetic field strength at cells and single axons levels. As such, an observing ego can recognize EM fields in its surroundings by either strength, scope, or changes in FoK-FIP. It responds through reactions to the general presence of an EM field by sensing strength, scope, or changes in specialized small magnetic signals and through experiencing four phases of a narrowed range of interoceptive signals.The idea that an observing ego can do this implies that cognition carries a magnetic property susceptible to being influenced by electromagnetic forces generated by EM fields. This concept is congruent with a theory that models the nervous system’s inhomogeneous electromagnetic field and includes “electromagnetic receptors” on cells (see Isakovic et al., 2018). The FoK-FIP theory builds on the “electromagnetic receptor” concept in which an observing ego’s sensitivity to FoK-FIP ultimately is how the signal centering through the biological node “bends back on” to affect an overall self-reflective or “electromagnetic receptor-like” cognitive process. When correlated with a living organism model FoK-FIP generate:

1. Specialized homogenous EM fields consisting of restricted intracellular oscillatory magnetic fields with a uniform composition (i.e., the amount and type of oscillations are the same). These specialized EM fields produce homogeneous EM fields through the feedback loops mechanism.

2. Specialized inhomogeneous EM fields consisting of a mixture of restricted intracellular oscillatory magnetic fields whose composition is not uniform (i.e., consists of two or more phases). These specialized EM fields produce inhomogeneous EM fields through the feedback mechanism.

### FoK-FIP, mental stability, and a sense of wellbeing

This FoK-FIP theory places mental stability as the central construct that gives rise to a sense of wellbeing, with stability itself defined through FoK-FIP. This theory includes a developmental theory that builds on the metacognition and prosociality/impulse control literature. It consists of a reaction schema that distinguishes between reflexive/habitual/volitional reactions that provide parallelism between the internal representation and the behavioral response. An observing ego is posited to make decisions through goal-directed reactions while cognitively broadcasting. Further, an observing ego has relatively little direct access to interoceptive signals. Instead, an observing ego is linked to interoceptive signals through feedback loops with the biological node, FoK-FIP. Through FoK-FIP, an observing ego intervenes in response to emerging signals as either an “impulsive reaction” or a “reaction with forethought.” Mental stability changes as a function of the observing ego’s volitional reactions. When an observing ego emerges through FoK, it begins to broadcast with an automatic response pattern to FoK-FIP cognitively. In this process, reflexive reactions lead to habitual reactions which lead to volitional (also intentional or deliberate) reactions capable of producing cognitive broadcasting effects:

1. Reflexive reactions: An unconditioned response with an assumed degree of automaticity and a high degree of reflexivity. Reflexive reactions create an observing ego’s intuitive sense.

2. Habitual reactions: A combined response as a reflexive reaction followed by an impulsive reaction (i.e., a sudden reaction without planning and impulse action). Habitual reactions represent an over-practiced response experienced by an observing ego with a high degree of having little mental choice due to reflexivity.

3. Volitional reactions: A conditioned response with a degree of agency. An observing ego decides on and commits to a particular course of action through volitional reactions. Things are perceived as if they were caused to happen. There are two volitional reactions: An impulsive reaction and a reaction with forethought.

This FoK-FIP theory may add to the discussion surrounding the concept of “free-will” that in this theory relates to an observing ego that is free to respond but constrained in its goal-directed reactions by varying degrees of FoK-FIP sensitivity. An observing ego’s volitional control may require changing its relationship to FoK-FIP that center its sensations or feelings related to interoceptive signals (i.e., signals of the body’s internal state). Interoceptive awareness is an umbrella term that encompasses any (or all) of the different interoception features accessible to conscious self-report [15]. Congruent with a Buddhist psychological model (see Grabovac et al., 2011), feeling tones (i.e., pleasant, unpleasant, or neutral) represent an observing ego’s immediate and spontaneous awareness of FoK-FIP in which attachment and aversion can occur when they are pleasant or unpleasant. An observing ego’s attachment to or aversion to pleasant or unpleasant FoK-FIP results from reflexive and habitual reactions and the motivation to procure or avoid through cognitive broadcasting. The mindfulness literature has covered reacting versus responding; the novelty of this theory is the FoK-FIP-based feedback mechanism. Cognitive flexibility is broadly defined as the ability of an observing ego to change its relationship to the internal representation by sensing specialized small magnetic signals and experiencing four phases of a narrowed range of interoceptive signals. Success or failure to change this relationship through volitional reactions results in cognitive broadcasting as the behavioral response. An observing ego cognitively broadcasts with increased or decreased stability through volitional reactions. As such, this means it is more or less in control of how it defines interoceptive signaling. What dictates an increase or decrease in stability is the success or failure of an observing ego to anchor its reactivity to FoK-FIP ultimately. Attachment or aversion-driven cognitive broadcasting is a behavior of specialized interoceptive awareness to procure or avoid pleasant or unpleasant FoK-FIP ultimately. Through FoK-FIPs’ centering of signaling, posited is that mental instability of observing egos can occur. The mental stability of an observing ego correlates with the behavior of a closely related living organism, the three-dimensional model of interoceptive cognition.

Posited is that this FoK-FIP theory is congruent with psychological research in which greater pain relates to emotional stress and limited emotional awareness, expression, and processing [17]. In this theory, the single factor FoK-FIP acts as a unitary domain and a general process of understanding pain and emotion. The intent of this FoK-FIP theory, at a minimum, is to encourage clinicians working with patients who have persistent pain to at least inquire about – if not explore at length – a special type of interoceptive sensation in a region of the body rather than an organ as relates to emotion. This FoK-FIP theory builds upon theoretical models and improves upon other models by advancing how a mechanism of feedback loops interrelates the various executive subjective functions through an informational processing unit, FoK-FIP. In the subjective cognitive broadcasting process, the volitional reactions of an observing ego act as feedback to FoK-FIP, through which specialized interoceptive awareness senses specialized small magnetic signals. FoK-FIP center an observing ego’s sensations or feeling so that it acts as the magnetic sensor, with varying interoceptive sensitivity, which makes its goal-driven reactivity uncertain but sets the fundamental limit on the smallest external magnetic field sensed. The FoK-FIP theory posits that affective states arise from cognitive broadcasting of an observing ego and stands in contrast to theories of basic emotions which posit that the neural system subserves every emotion. The FoK-FIP theory posits that it represents a conceptual shift needed in the empirical approaches taken to studying emotion and affective psychopathologies. Further, the FoK-FIP theory may change established notions about electrical activity in living organisms, more consistent with many recent findings from behavioral, cognitive neuroscience, neuroimaging, and developmental studies of affect.

An observing ego is neither the FoK nor the FIP. However, when this viewpoint emerges and senses inhomogeneous specialized small magnetic signals followed by inhomogeneous phases of a narrowed range of interoceptive signals, it often becomes attached or aversive to FoK-FIP. Observing egos can sense inhomogeneous perturbation congruent with inhomogeneous EM fields occurring in the nervous systems of living organism models. Understood through living organisms’ models with brains and spinal cords, an inhomogeneous EM field generated through specialized biomagnetism exerts electric and magnetic forces on the central nervous system (CNS) cells. This process can influence cell migration and adhesion in pathologic conditions, including where cells have already penetrated the blood-brain barrier [16]. The FoK-FIP theory posits that it improves understanding of physiological and pathological processes that can open up novel therapeutic strategies based on EM field modulation or cell surface charge alteration. These therapeutic strategies include an observing ego inferred through living organism models’ behavior, and where human models can provide verbal feedback. Central to this theory is that nonconscious processes and automaticity can drive an observing ego through FoK-FIP. As such, an automatic response pattern process drives an observing ego’s understandings of interoceptive experience. Differences in interoceptive sensitivity of an observing ego determine the extent to which objective informational processing activities and reflexivity dominate the meaning an observing ego imposes on experience. This FoK-FIP theory is congruent with two different models (see Ekman, 1992) (see Posner & Russell, 2005). The discrete emotion and dimensional models [18] suggest six basic emotions: happiness, sadness, anger, fear, disgust, and surprise. In contrast, a two-dimensional scale of emotions [19] consists of the valance (i.e., positive and negative feelings) and arousal (i.e., the intensity of the feeling, either high or low). This FoK-FIP theory combines and modifies those models to classify the observing ego’s emotion to allow benchmarking of performance and simplicity of binary classification:

1. FoK arousal (low high range of 1–5): an observing ego emerges through FoK with varying arousal degrees. Reflexive reactions create an observing ego’s “FoK arousal.”
2. FIP valance: an awareness of an observing ego by sensing specialized small magnetic signals and experiencing four phases of a narrowed range of interoceptive signals. When it appraises these signals as pleasant or unpleasant, attachment or aversion may occur. In this FoK-FIP theory, emotions are physiocognitive states that include happiness, sadness, fear, anger, surprise, embarrassment, jealousy, guilt, and pride [20], in which there are core (also primary) and secondary emotions.
  1. Fear: A core emotion in which unpleasant FoK-FIP act as an antecedent to this physiocognitive state
  2. Happiness: A core emotion in which pleasant FoK-FIP act as an antecedent to this physiocognitive state.
  3. Secondary emotions (i.e., sadness, anger, surprise, embarrassment, jealousy, guilt, and pride) in which core emotions (i.e., fear or happiness) acts as an antecedent to these physiocognitive states.

FoK-FIP do not disappear; instead, an observing ego remembers specialized self-generated biomagnetism related activity in a way that includes four phases of a restricted range of interoceptive signals:

1. Nociception – A continuous specialized restricted range of oscillating magnetic fields results in a continuous narrowed range of interoceptive signals experienced by an observing ego with decreased tolerance to pain.
2. Antinociception – A continuous specialized restricted range of oscillating magnetic fields results in a continuous narrowed range of interoceptive signals experienced by an observing ego with increased tolerance to pain.
3. Disgust – A rush of a specialized restricted range of oscillating magnetic fields results in an observing ego experiencing an unpleasant rush of a specialized narrowed range of interoceptive signals that quickly subsides.
4. Orgasm – A rush of a restricted range of oscillating magnetic fields results in an observing ego’s experiencing a pleasant rush of a specialized narrowed range of interoceptive signals that quickly subsides.

Congruent with the fact that feedback loops occur through interactions between cells or cells and nerve fibers, FoK-FIP act as an informational processing unit. In this process, FoK-FIP regulate the strength of centering that affects the objective mind (i.e., interoceptive signals) and the subjective mind (i.e., observing ego). Through living organism models with central nervous systems (i.e., brains and spinal cords), FoK-FIP are DNA encoded intracellular events in which all major processes in the nervous system depend. The biological node acts as the central or connecting point where lines or pathways of oscillating magnetic fields intersect or branch. This process includes an observing ego connected with interoceptive signals and interoceptive systems. As such, specialized interoceptive awareness through feedback loops with FoK-FIP connects to systems that include a complex array of signaling (e.g., the endocannabinoid system and the autonomic nervous system). When correlated with a living organism model with a brain and spinal cord, it is understood that that FoK-FIP center physiological signaling using the sympathetic component of the autonomic nervous system (ANS). FoK-FIP cause an observing ego’s emergence and automatic response pattern. Intracellular FoK-FIP result in an observing ego that acts as an excitatory cognitive process. Through specialized biomagnetism, depolarization causes the interaction between cells and nerve fibers. The feedback loops between FoK-FIP and interoceptive signals occur. When specialized biomagnetism depolarization occurs, it is understood that an observing ego connects with the sympathetic component of the ANS. Through specialized biomagnetism depolarization understood due to a living organism model, a specific type of interoceptive sensation occurs in a region(s) of the body rather than an organ.

Through a relationship with the model, an observing ego’s reflexive and habitual reactions are congruent with an activation of the sympathetic (i.e., “fight or flight”) component of the ANS. In this scenario, an observing ego aims to define interceptive signals (i.e., signals of the body’s internal state) to increase movement and strength using FoK-FIP to broadcast cognitively. Understood through the model, a closely related living organism (e.g., human or another animal) could be in a stressful situation that threatens survival, exercising, or in the moments just before waking. In contrast, volitional reactions, such as when an observing ego’s impulsive reaction is followed with a reaction with forethought, are congruent with an activation of the parasympathetic (i.e., “rest and digest”) component of the ANS. In this scenario, an observing ego aims to define signals to function in opposition to sympathetic nervous stimulation through cognitive broadcasting. Nevertheless, understood through the relationship with the model, an observing ego using volitional reactions cannot exert significant control over vascular tone. An observing ego through a reaction with forethought can engage in deliberate and thoughtful cognitive broadcasting through a willingness to go toward a narrowed range of interoceptive signals. This volitional reaction after emotion or mood anchors an observing ego’s reactivity to FoK-FIP and can result in feedback that changes signaling centering occurring through the biological node. Further, an observing ego is a perceiver of signals which can always find its way back to FoK-FIP by sensing specialized small magnetic signals and experiencing four phases of a restricted range of interoceptive signals. An observing ego’s goal-directed response that includes detecting FoK-FIP and going toward them suppresses its urge after an emotion or mood to avoid or procure FoK-FIP. This process is understood through a human model where an observing ego’s willingness to anchor its reactivity to FoK-FIP is congruent with mindfulness to change the relationship to a specific type of interoceptive sensation in a region of the body rather than an organ. In this process, signaling occurring in a bottom-up manner is interrupted, which prevents signals that would otherwise activate such structure as the amygdala (a brain structure involved in emotion).

### FoK-FIP and interoceptive immersion

This FoK-FIP theory builds upon the amply recognized literature that metacognitive and self-regulatory abilities are of fundamental significance for children’s general and academic development [21]. The FoK-FIP theory posits that it improves upon other developmental models with the biological node and feedback loop mechanism where metacognitive and self-regulatory subjective activities occur through an observing ego’s cognitive broadcasting. In this FoK-FIP theory, a developmental model is included with three interoceptive “maturity” levels (i.e., immersion, engrossment, and engagement). As previously discussed, when an observing ego cognitively broadcasts, it creates a world that it projects around itself. Through embodied states, FoK-FIP connect the subjective mind, an observing ego, with the objective mind, interoceptive signals. This FoK-FIP theory does not include scientific materialism in which causal reduction of mind to brain affirms matter as the fundamental entity or property [22]. This theory envisions a “self” as an observing ego, a cognitive force, a subjective viewpoint, and a phenomenon harder to empirically prove because it relates to sentience or awareness emergence. Observing egos rather than living organism models are posited closest to representing the image of the mind without beginning or end. In the subjective cognitive broadcasting process, an observing ego, to varying degrees, will define signals to create two types of perception (i.e., egocentric or altruistic). Egocentric or “selfish” perception (thinking of only oneself) and altruistic or “generous” perception (thinking of others, primarily including their wellbeing) reveal the relationship between an observing ego and FoK-FIP ultimately. Further, both perceptions fundamentally are viewpoints that reveal degrees of an observing ego’s sensitivity to FoK-FIP and its willingness to override an over-practiced response. An observing ego’s performance on any cognitive broadcasting task is congruent with its volitional reactions to competing influences in which FoK-FIP center its sensations or feelings. The FoK-FIP theory may help advance understanding related to social research regarding the potential importance of emotional communication, empathy, attachment, and rejection [17]. Understood through the nervous system of a living organism model, FoK-FIP are DNA encoded intracellular events in which specialized electromagnetic (EM) fields (e.g., homogeneous or inhomogeneous) occur. Through cell and nerve fibers interactions, specialized EM fields are congruent with EM fields in the nervous system representing increasingly influential conscious representational systems. These systems influence posited to be developed through specialized EM fields generated by FoK-FIP. Further, because self-generated specialized biomagnetism centers on observing ego’s sensations or feelings, homogeneous EM fields in the nervous system act like a normal state and inhomogeneous EM fields act as a pathologic state. As such, specialized EM fields represent competing influences that can affect the development of an observing ego’s attention and integration of competent subjective executive functions while cognitively broadcasting. This process is understood through three levels of interoceptive immersion:

1. Interoceptive total immersion: represents the highest level of a cognitive broadcasting subjective process understood through animal models that often occurs at birth and includes a lack of empathy. Empathy is defined here as an observing ego understanding and sharing another’s feelings; that is not the situation with total immersion. Instead, at this level, an observing ego has “current concerns” that are primarily nonconscious FoK-FIP driven, and they do not challenge their egocentric perceptions. This sort of thinking manifests through cognitive broadcasting, including higher-end cognition in which an observing ego perceives its close relationship and believes itself to be a model of interoceptive cognition consciousness, a living organism. As such, an observing ego with vague certainty can know that only the self matters through varying degrees of deriving its sense of self that may include a three-dimensional “object” that walks, talks, and eats after emerging through higher-end cognition.
2. Interoceptive engrossment: represents the impulsivity, sensation-seeking, level of a cognitive broadcasting subjective process understood through animal models that often occurs during adolescence [23] and includes a mix of empathy and apathy. Apathy is defined here as an observing ego’s lack of interest, enthusiasm, or concern regarding understanding other viewpoints. At this level, FoK-FIP center sensations or feelings so that an observing ego cognitively broadcasts intending to seek pleasant and avoid unpleasant specialized small magnetic signals and a narrowed range of interoceptive signals. An observing ego perceives this impulsivity trait to varying degrees through its close relationship with the living organism model. With an assumed degree of automaticity and a high degree of reflexivity, an observing ego in relationship with an animal model can experience emotions/moods (to varying degrees). An observing ego highly attached or aversive to sensations or feelings seems to bond with FoK-FIP through an over-practiced response. As such, it can experience engrossment, a pure mental-like state with a high degree of having little choice. At this level, an observing ego is often less aware of being the “invisible” observer and has a “valanced relationship” with the animal model, especially when the model is human, consisting of feelings (i.e., positive or negative). As such, this engrossment level includes beliefs about being this living organism, which FoK-FIP acts as an antecedent to core emotions (i.e., happiness or fear) that often result in a cascade of mental events. An observing ego with heightened sensitivity to FoK-FIP will find it more difficult to react with forethought and this will restrict its ability to control impulsivity while cognitively broadcasting. It will understand perception through its relationship with the closely related model of higher-end cognition in which a good deal of risk-taking occurs during adolescence, including some of the most hazardous forms of behavior [23].
3. Interoceptive engagement: represents the ability to overcome preference to FoK-FIP level of a cognitive broadcasting subjective process understood through enlightened courageous human models and other animal models (e.g., empathetic bonobo great apes, an endangered species) that often occurs during adulthood and includes an abundance of empathy with an absence of apathy. An observing ego enters this level by overcoming preference barriers that arise through sensing specialized small magnetic signals and experiencing four phases of a narrowed range of interoceptive signals. Accordingly, an observing ego needs to invest time, effort, and attention in learning how to use its volitional reactions (impulsive reaction only or impulsive reaction followed by reaction with forethought). Doing this can overcome FoK-FIP related activities that act as an antecedent to core emotions (i.e., fear or happiness). In this process, an observing ego becomes cognitively self-conscious as part of the experiential component of this process in which there is no “object” of higher-end cognition to support representation, but with vague certainty, it knows sentience or awareness, congruent with “Cogito, ergo sum” (“I doubt, therefore I think, therefore I am,” René Descartes). When an observing ego has FoK-FIP sensitivity and willingly anchors its reactivity, posited is this contributes to metacognitive and self-regulated performance. Through goal-directed reactions, the willful, impulsive reaction followed by a reaction with forethought sequence represents an observing ego’s skillful mindfulness training that changes its relationship to FoK-FIP. Due to subjective processes, an observing ego can be aware of itself as the invisible viewpoint of interoceptive cognition consciousness while perceiving its close relationship to a living organism model. Because cognitively broadcasting differently, the living organism model becomes a visual indicator that provides feedback to an observing ego of its ability to anchor its reactivity to FoK-FIP ultimately. Further, an observing ego can significantly impact how it perceives the physical world and defines it through skilled cognitive broadcasting. Cognitive broadcasting’s fundamental subjective significance occurs when an observing ego can anticipate FoK-FIP and decides to commit to a particular volitional reaction as goal-directed action to change its relationship to FoK-FIP willfully. Understood through the human model, mindfulness techniques in modern medicine and healthcare are highly teachable, and later in this article related research in which a FoK-FIP mindfulness technique is presented.

## Discussion

Change can be discussed scientifically in many different ways. Nevertheless, posited is that change represents aspects of the mind in which one event, process, state, or object all relate to this fundamental entity. The Big Bang is the standard theory in which space, time, matter, and all energy currently in the Universe formed, which, based on the rate of expansion and the distance of galaxies, may have occurred some 10 to 15 billion years ago. The theory was originally formalized by a Belgian Catholic priest, mathematician, astronomer, and professor of physics Georges Henri Joseph Édouard Lemaître, and this evolved into the Big Bang theory via observations and theoretical consideration. Through expanding matter from a state of extremely high density and temperature, conditions have progressed from a hot early Universe to one in which the gas within it has cooled. When the radiation that filled the Universe as scattered change (that included light) traveled without being scattered, the Universe became transparent. Currently, the Universe is filled with radiation, the remnant of heat termed cosmic microwave background (CMB). As such, conditions that began with the rapidly cooling Universe formed “simple matter” in which, as the Universe expanded, caused the formation of galaxies, stars within galaxies, and the Earth. The Earth cannot be older than the solar system and is perhaps between 4.4 and 4.57 billion years old. The Earth’s magnetic fields, electromagnetic (EM) waves, and the force of gravity posited are significantly related to interoceptive cognition consciousness congruent with the emergence of observing egos. Further, the history of the physical world occurs when two different processing activities, subjective and objective, are connected by FoK-FIP through embodied states. This concept is understood through living organisms models evolving since the Earth’s formation:

1. The geomagnetic field (GMF), a magnetic field that surrounds the Earth and extends from the Earth’s interior out into space, is not fully understood. The GMF may have existed for at least 3.5 billion years [24]; another mechanism drove a magnetic field before. Understood through geophysical theorizing, the GMF results from electrical currents generated by the movement of molten iron and nickel within Earth’s outer core producing the dynamo effect. Accordingly, a celestial body like Earth can generate a magnetic field through a self-exciting dynamo mechanism. This dynamo effect is a self-sustaining process in which an electric current produces a magnetic field that interacts with a fluid motion to create a secondary magnetic field. In this process, a rotating, convecting, and electrically conducting fluid maintains a magnetic field over astronomical time scales. Through models, it is understood that the GMF is an unavoidable environmental factor for all living organisms with DNA and tissue. The magnetic field through the dynamo effect can change quickly (e.g., within an hour in magnetic storms). Additionally, the GMF is primarily dipolar with north and south magnetic poles.
2. Electromagnetic (EM) waves travel from the Sun to the Earth across space and provide all the energy that supports life in which light is part of the spectrum of electromagnetic energy. This energy travels as waves at the speed of light and consists of varying wavelengths (i.e., the distance between successive wave crests). In contrast, EM waves can vary in frequency (i.e., the oscillation’s rate occurrence and constitutes a wave). Electromagnetic energy has a spectrum that includes (from longest wavelength to shortest): radio waves, microwaves, infrared, optical, ultraviolet, X-rays, and gamma-rays..
3. The force of gravity gets weaker farther from the center of the Earth. Understood through models, since life first began on Earth, gravity’s force has been ever-present, and slight variations can significantly impact living organisms with DNA and tissue. Since the first single-celled organism emerged on Earth, gravity has affected the development of life that has evolved to rely upon and cope with the force of gravity in various ways. For example, in stronger gravitational fields, the size of cells decreases, and in weaker gravitational fields, the size of cells increases. Understood is that gravity is a limiting factor in the growth of individual cells in which the size of the cell is inversely proportional to the strength of the gravitational field exerted on the cell. Through the Sun’s gravity, the Earth orbits at a distance, and living organisms require energy to survive in which the Sun provides light and warmth.

Relationships that began billions of years ago are posited through embodied states between observing egos and interoceptive signaling in the physical world. Further, the cognitive broadcasting of subjective minds was primitive through embodied states, and the objective minds consisted of a few electromagnetic energy emitters in which FoK-FIP centered relatively simple weak magnetic fields. Understood through single-celled organism models is that interoceptive cognition consciousness began with similar origins and evolved. For approximately the past 3.5 billion years, biological processes have caused an oxygen-rich atmosphere. As such, life as we know it emerged and evolved on Earth that began with the earliest life forms as single-celled or unicellular prokaryotic organisms such as bacteria. This single-celled organism lacking a nuclear membrane-enclosed nucleus led to the emergence of many millions of species. Accordingly, eukaryotic models emerged on the world scene with a nucleus enclosed with a nuclear envelope along with all the protists (i.e., any eukaryotic organism that is not an animal, plant, or fungus).

In general, members of eukaryotic organisms include:

1. Fungi (e.g., yeasts, rusts, smuts, mildews, molds, mushrooms).
2. Fungus-like organisms (e.g., slime molds).
3. Plants as multicellular and photosynthetic eukaryotes.
4. Animals are considered multicellular eukaryotic organisms that in most cases consume organic material, breathe oxygen, move and reproduce sexually.

### FoK-FIP and the components map model with interoceptive markers (IMs)

This model is used as a framework for conceptualizing cognitive broadcasting by an observing ego that is a subjective process. It differs from current thinking, with FoK giving rise to an individual (i.e., an observing ego) who can define interoceptive signals using FoK-FIP. Through cognitive broadcasting an observing ego creates a world that it projects around itself. This model posits that it can improve the biologically inspired (i.e., bio-inspired) computing optimization algorithms to effectively solve scientific and medical problems. It represents a novel approach based on principles and inspiration of the biological evolution of nature. Included in this model are interoception, proprioception, and exteroception that can potentially add to existing schemas. Cognitive broadcasting is not a simple process but instead has several facets that occur when an observing ego senses, interprets and integrates information about the state of inner body systems [15]. Interoceptive attention, detection, discrimination, accuracy, insight, sensibility, and selfreport [15] are posited to be congruent with an observing ego’s perception during cognitive broadcasting. Further, none of these different elements of cognitive broadcasting could occur without FoK-FIP. As previously discussed, this FoK-FIP theory posits that FoK, “awareness charge,” causes the emergence of “cognitive force,” an observing ego, and FIP enables specialized interoceptive awareness to sense specialized small magnetic signals and experience four phases of a narrowed range of interoceptive signals.

An observing ego acts as a viewpoint and is not interoceptive cognition consciousness itself; rather, it is the subjective mind that cognitively broadcasts using FoK-FIP. Cognitive broadcasting occurs through an observing ego connected to FoK-FIP that centers its sensation or feelings. Through reactions producing both automatic and deliberate responses, the subjective mind interacts with the objective mind (i.e., interoceptive signals), and an observing ego defines conscious events. As such, cognitive broadcasting is a subjective process that includes an observing ego reacting (e.g., reflexively, habitually, and volitionally) where volitional reactions represent goal-directed actions and how an observing ego’s decisions are promoted or prevented. In the subjective cognitive broadcasting process, an observing ego creates to varying degrees nonconceptual and conceptual mental content that allows it to express its intuitions as best possible about the reality it perceives:

1. Nonconceptual mental content is defined as a cognitive broadcasting process that creates thinking based on the theory that some mental states can represent the world of appearances even though an observing ego possessing those states does not have the concepts required to specify their content [25]. Thinking of this sort may be summarized as free from conceptual limitations resulting from entertaining subject and object concepts [26].
2. Conceptual mental content is defined as broadcasting that creates thinking based on abstract cognitive processes. As such, it is congruent with ideas rather than events that include fundamental units of thought (e.g., true or false, believed, desired, hoped for) from an observing ego’s perspective [25]. Included in this process is conceptualization, or the process of forming a concept of something that includes analytical or problem-solving ideas and symbols (a mark, word, or sign that, through a mental process, links an idea, ‘object,’ or relationship).

**Figure 2.**
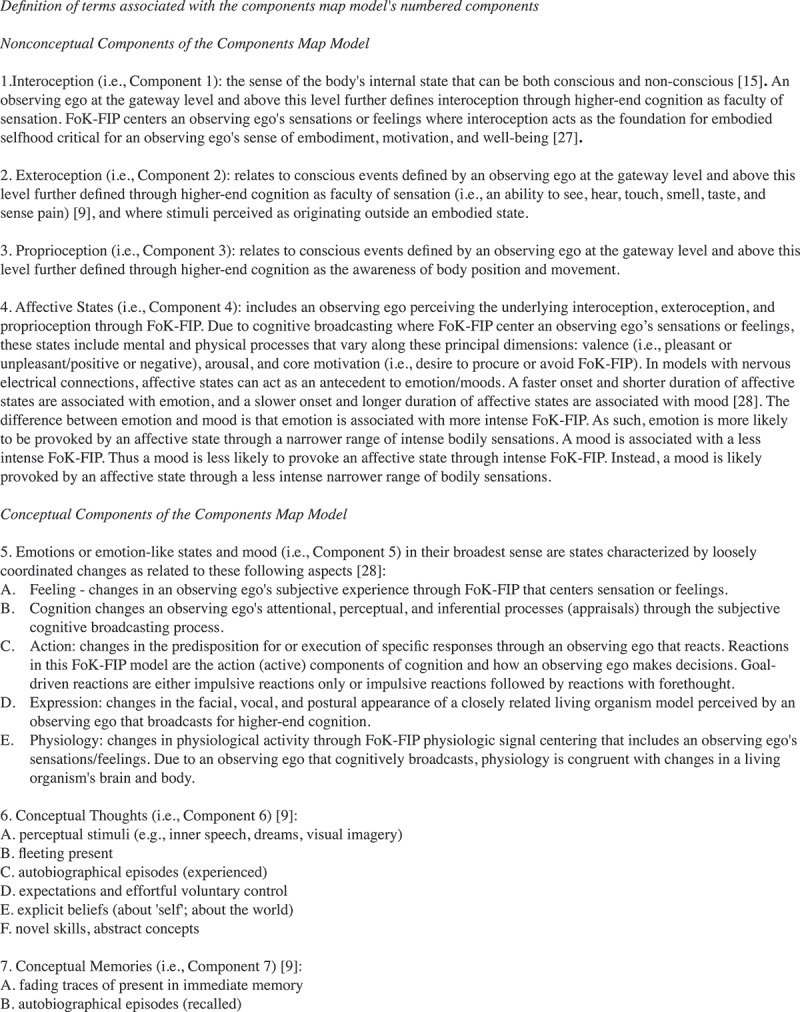
Terms of the components map model with interoceptive markers (IMs).

Observing egos explain how information gets processed so that the physical world includes a particular activity, interest, or mental experience (e.g., touch, feel, sense, measurement, or detection) that occurs when specialized interoceptive awareness senses FoK-FIPs and differentiates signals. This process can be envisioned through the components map model with IMs. This model sets out the different layers of cognitive processes through numbered components with interoception acting as a substrate of mental content. Numbered interoceptive markers (IMs) are included in this model to improve the study of cognitive broadcasting and thus improve upon other interoceptive cognition models. Definition of terms is provided congruent with the model’s numbered components [Figure 2] and [27,28]. Each numbered component represents a subsystem (i.e., a self-contained system within a more extensive system) with causal relationships in which one subsystem can affect other subsystems and a ‘ripple effect’ can spread through the whole system. A chain of cause-and-effect in this model consists of cognitive cycles represented by the numbered components in a series or sequence of recurring mental events. With each cognitive cycle, an observing ego senses the current situation and interprets it regarding ongoing interoceptive goals. The events of cognitive broadcasting represent the constant change that may be unpredictable. However, change is constrained through FoK-FIP. In this model, components 1–3 represent the first stages of the cognitive broadcasting process, which concerning an observing ego’s perspective are nonconscious processes (i.e., akin to preconscious events). Components 4–5 represent the gateway level, stages further along in the cognitive broadcasting process where information becomes more understandable to an observing ego through affective states and related rudimentary mental content (i.e., involving basic ideas). At the gateway level, an observing ego with sentience or awareness vaguely related to nonconceptual thought processes knows that it exists through FoK-FIP ultimately.

Definition of terms associated with the components map model’s numbered components

Cognitive broadcasting at the gateway level represents automatic subjective processing. In this process interoceptive signals are defined by an observing ego using FoK-FIP that centers its sensations or feelings. An observing ego has little access to intervene in how interoceptive signals get broadcasted at the gateway level in which nonconceptual mental content is created. An observing ego responds with reflexive reactions and perceives signaling at the gateway level through sense-impression. Perceived by an observing ego through an unelaborated elementary awareness of stimulation are interoception (i.e., signals of the body’s internal state), proprioception (i.e., sense of movement and position), and exteroception (i.e., sight, sound, smell, touch, taste, and pain sensation). Importantly at the gateway level, an observing ego can become more or less attached or aversive to FoK-FIP that center its sensations or feelings through four phases of a narrowed range of interoceptive signals occurring as affective states (i.e., component 4). An observing ego perceives FoK-FIP at the gateway level through degrees of affective feelings (i.e., nociception, antinociception, disgust, or orgasm). Through this subjective cognitive broadcasting process, an observing ego can experience attachment or aversion to FoK-FIP that act as an antecedent to emotions/moods (also emotion-like states and moods) (i.e., component 5). After perceiving emotion/moods (i.e., component 5), an observing ego responds with varying degrees of impulsive reaction based on its sensitivity to FoK-FIP. As such, higher-end cognition occurs only later in the cognitive broadcasting sequence through conceptual thoughts (i.e., component 6) or conceptual memories (i.e., component 7). Importantly when an observing ego broadcasts for higher-end cognition, varying degrees of the reality of “others” can occur. Additionally, the cognitive broadcasting of an observing ego above the gateway level is envisioned as specialized interoceptive awareness entering the “theater district,” a part of subjective processing that produces “mental movies.” The close relationship between an observing ego and the living organism model plays a starring role in this process. Through this relationship, an observing ego perceives the model with various degrees of selfhood in which the living organism causes more or less of an observing ego’s sense of identity. The living organism’s behavior is congruent with the relationship between the subjective mind and the objective mind. Through cognitive broadcasting, an observing ego with degrees of FoK-FIP sensitivity causes signals to be defined with more or less empathy or apathy:

1. Empathy: the ability to understand and share the feelings of another.
2. Apathy: a lack of interest in, enthusiasm for, or concern for another’s feelings.

**Figure 3.**
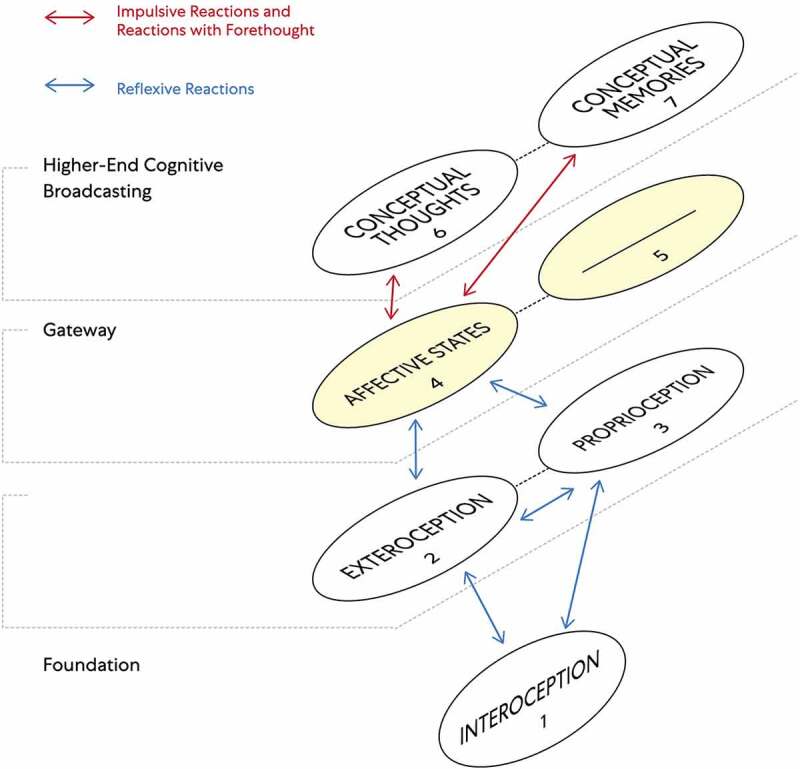
The components map model with interoceptive markers (IMs) congruent with an observing ego in close relationship to a living organism (e.g., plants and slime mold) with non-nervous electrical connections.

**Figure 4.**
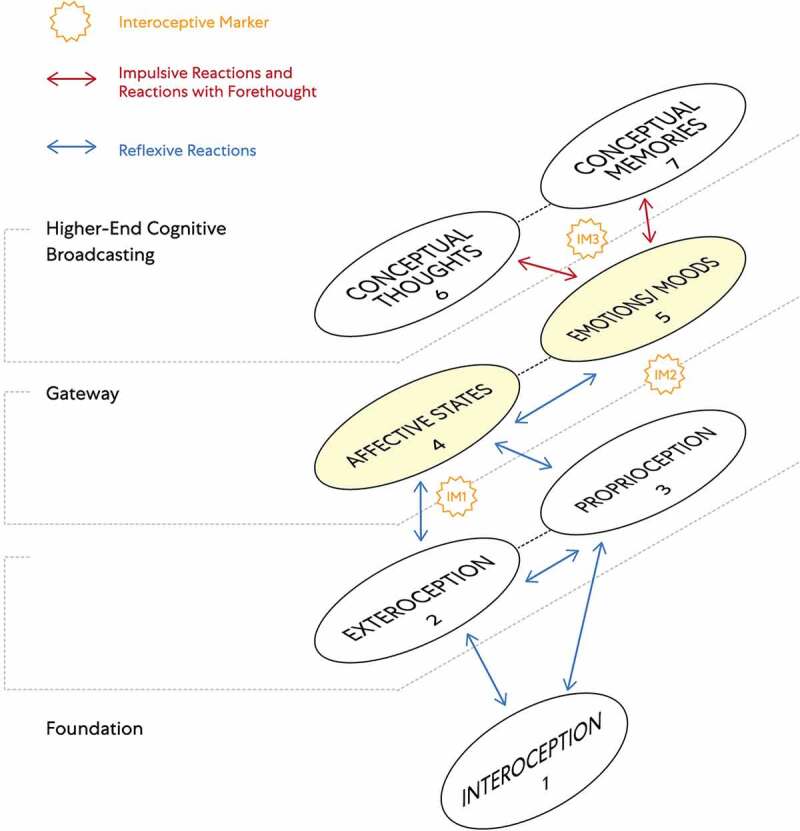
The components map model with interoceptive markers (IMs) congruent with an observing ego in close relationship to a living organism (e.g., humans and other animals) with nervous electrical connections.

Understood is that FoK-FIP is DNA encoded intracellular events when correlated with living organism models. The living organisms are posited to be models of interdependent embodied relationships between subjective minds (i.e., observing egos) and objective minds (i.e., interoceptive signals) in a shared physical world. Further, two different components map models with IMs accommodate two living organisms models: Living organisms (e.g., plants and slime mold) with non-nervous electrical connections [Figure 3] and living organisms (e.g., humans and other animals) with nervous electrical connections [Figure 4]. When observing egos began to broadcast for higher-end interoceptive cognition posited, they began identifying with the models, living organisms. Further, we often believe ourselves to be the unique living organisms of the physical world when in reality, we are unique observing egos. As such, we are nothing more or less than the same kind of different. This idea is congruent with major philosophical works, including Buddhist roots [5], traditional philosophers, and contemporary scholarship that melds science with embodied selfhood. This FoK-FIP theory includes observing egos with a measurable dimension of variation. To illustrate this concept, the author represents an observing ego that cognitively broadcasts and thus perceives mental content, including logical and spatial competencies of varying degrees in a unique relationship with a human model. In contrast, an observing ego in the state of waking consciousness is currently reading this article and is also in a unique relationship but with a different human model. Although there is a difference between an observing ego, the author, and the reader, posited is that our emergence has the same FoK-FIP mechanism but a different FoK origin. Further, because of observing ego’s relationship to models, the definition of consciousness has several relative meanings. In biomedical science, the terms “conscious state” or “waking state” means the state of waking consciousness with subjectivity (e.g., responsiveness to questions, commands, and mild pain; by the classical scalp EEG of waking, and by the ability to describe oneself and current events) [9]. As an experimental variable, consciousness refers to the dimension of consciousness versus unconscious brain events in a way that allows the study of brain differences attributable to consciousness. This definition involves a measurable dimension of variation and differs but correlates with the first definition (i.e., waking state) [9].

This model sets out the different layers of cognitive processes of interoceptive cognition consciousness through numbered components. An observing ego broadcasts cognitive cycles consisting of a series of numbered components linked. Importantly emotions/moods are not part of the subjective cognitive broadcasting process with non-nervous electrical connection models.

This model sets out the different layers of cognitive processes of interoceptive cognition consciousness through numbered components. An observing ego broadcasts cognitive cycles consisting of a series of numbered components linked. Numbered IMs are included in this model to improve the study, diagnosis, prognosis, or treatment of interoceptive psychopathology: IM1 -Affective feelings, IM2 -Emotion or mood, IM3 - Impulse/Urge

An observing ego may mistakenly assume a unitary self-process underlying experience through the subjective cognitive broadcasting process. Nevertheless, an observing ego’s “selfhood” unfolds across many partially independent and partially overlapping levels of description. Research with experimental manipulations and clinical conditions shows that this selfhood may be teased apart in the laboratory or fall apart during neuropsychiatric illness [13]. Research has shown that many features of cognition aim to predict the meaning of bodily sensations. This FoK-FIP theory is congruent with other models but differs in that interoceptive magnitude refers to the perceived intensity of the sensation [29] through an observing ego that cognitively broadcasts using FoK-FIP. Interoceptive attention is posited to refer to an observing ego in which FoK-FIP centers its sensations or feelings to interoceptive signals. Further, an observing ego connected to a biological node (i.e., FoK-FIP) explains how interoceptive awareness includes integrated, abstracted, and interpreted representations of the current embodied state [30]. FoK-FIP acts as a “searchlight” of attention [31]that plays a prominent role in specialized interoceptive awareness through an observing ego. This FoK-FIP theory, in a novel way, adds to the discussion of the “Binding Problem,” a famous continuing question in computational neuroscience that concerns how items are encoded by distinct brain circuits representing a mind-body problem [32]. Recent evidence shows interoceptive ability depends upon the interoceptive signal to be perceived [33]. This process envisioned through the components map model with IMs contributes to a novel understanding of animal-based competing techniques that could guide future endeavors related to novel interdisciplinary research. For example, using human models with verbal feedback, sensitive magnetometers might quantitate the mental stability of an observing ego by measuring electrical activity in the brain congruent with EM fields in the nervous system (e.g., homogeneous or inhomogeneous). The aim of this novel research includes: (a) examining whether specialized self-generated biomagnetism relies on a system of brain regions that are important for signal centering and constituting a “FoK-FIP network” (b) ascertaining whether or not dysfunction signal centering through FoK-FIP underlies different mental health conditions including their effect on emotional dysregulation of observing egos, and (c) examining the utility of FoK-FIP in understanding the neurobiology of fear. When the model is of the “with nervous electrical connections” type (e.g., human or another animal), the volitional responses of an observing ego occur after a reflexive reaction is transformed to an impulsive reaction following emotions or moods (i.e., component 5). Importantly, when the model of interoceptive cognition does not have a brain, emotions/moods are not included as a component of interoceptive cognition consciousness.

The components map model with IMs is an algorithm and alternative method for understanding behavior in which feedback loops between an observing ego and FoK-FIP, the biological node, is critical to developing solutions to complicated problems by stimulating analogic reasoning and thinking. The components map model with IMs understood through animal models [34]is posited to be congruent with nine optimization algorithms in the literature, based only on the biological behavior of some animals in fighting for food and mates (see Darwish, 2018). FoK-FIP centers an observing ego’s sensations or feelings so that cognitive broadcasting supports the body’s physiological condition [35]. This process includes an observing ego’s mental stability that changes as a function of its volitional reactions while it perceives feelings such as hunger and thirst and feelings of sexual arousal. In this FoK-FIP theory, an observing ego derives a nonconceptual “sense of self,” core motivation, and core emotions through FoK-FIP that occur at the gateway level:

1. The nonconceptual “sense of self”: a feeling of knowing one exists without conceptualizing how one exists.
2. Core motivation (to varying degrees): the desire to procure or avoid FoK-FIP (i.e., pleasant or unpleasant) as specialized small magnetic signals and four phases of a narrowed range of interoceptive signals.
3. Core emotions (or emotion-like states): fear and happiness as primary physiocognitive states.

Through broadcasting for higher-end cognition, the nonconceptual “sense of self,” core motivation, and core emotions result in core themes that occur above the gateway level. Core themes are posited to be the central part of higher-end cognitive broadcasting and the foundation for an observing ego’s further knowledge and understanding (to varying degrees). The core themes through subjective processing can be summarized as:

1. Food
2. Fluid
3. Sex.

Due to an observing ego’s close relationship with a model, in which the subjective mind (i.e., an observing ego) and objective mind (i.e., interoceptive signals) are connected by FoK-FIP, a living organism (e.g., woman, man, or another animal) can represent a “vision” of selfhood (i.e., the “I” and the “me”). The selfhood process is posited to occur through the subjective mind where the nonconceptual “sense of self” (occurring at the gateway level) through cognitive broadcasting gets transformed into aspects of the conceptual self. Through conceptual thoughts (i.e., component 6) or conceptual memories (i.e., component 7), displayed are “mental movies” perceived by an observing ego that to varying degrees are shaped through its relationship to the model as core themes:

1. The maintenance of life and growth (i.e., through food).
2. The homeostasis of body fluid regulation (i.e., through fluid).
3. The reproductive function perhaps as an evolutionary strategy to pool resources (i.e., through sex).

### FoK-FIP related research

The FoK-FIP theory represents modeling congruent with the relationship between stress and disease that is now well established but was not consistently recognized. In physics, the term ”stress” refers to the interaction between a force and the resistance to counter that force, and Hans Selye (1907–1982) first incorporated this term into the medical lexicon to describe the “*nonspecific response of the body to any demand”* [36]. Selye (i.e., the ‘father of stress research’), unlike others before him, focused on universal patient reactions to illness rather than the study of specific disease signs and symptoms. This FoK-FIP theory was created with similar intent in which stress refers to an observing ego using FoK-FIP to broadcast cognitively with implications of Quantum Mechanics. Observing egos react to FoK-FIP and thus can define interoceptive signals (i.e., signals of the body’s internal state) through cognitive broadcasting (to varying degrees). The laws of quantum mechanics determine the ultimate sensitivity of any measurement, and sentience or awareness emergence can relate to stress. An observing ego’s emergence from FoK can be stressful and a process understood when specialized interoceptive awareness has a higher-end cognition relationship with an animal model. The novelty of the FoK-FIP theory is that it correlates sentience or awareness with an observing ego that emerges with FoK-FIP sensitivity through specialized small magnetic fields. As such, FoK-FIP create a new understanding that opens avenues for novel empirical research. Emotional stress relates to fluctuations in small magnetic fields linked with electrical activity that may be empirically measured with new technologies (e.g., atomic magnetometer). Further using a human model, this measurement can be subjectively correlated through verbal feedback of FoK-FIP congruent with a specific type of interoceptive sensation occurring in a body region. In this way, FoK-FIP may add dimension to the suspected comorbidity aspect of interoception in disease widely discussed in the literature. Additionally, FoK-FIP may add to Selye’s work and stress research. FoK-FIP may impact scientific and lay communities alike in fields as diverse as endocrinology, complementary medicine, animal breeding, and social psychology [36].

Through FoK-FIP that occur in four phases congruent with small magnetic signals, an observing ego experiences (e.g., nociception, antinociception, disgust, and orgasm). An observing ego uses FoK-FIP to center its reactivity and response to the interoceptive signals it senses and integrates through the cognitive broadcasting process. In this process, FoK-FIP represents an observing ego’s intuitive feelings that may not be rationally understood but experienced as vague and with a sense of certainty. As such, FoK-FIP importantly differs from more basic interoceptive experiences (e.g., hunger, thirst, respiratory and cardiac signals). The subjective mind of an embodied state is an observing ego, and posited is that FoK-FIP is fundamental to its feelings and self-awareness. Further, fear as a core emotion cued through affective states is profoundly associated with FoK-FIP and can affect an observing ego’s perceptions of danger. This perception can lead to an inaccurate understanding of the physical world as a chronically dangerous environment. An observing ego’s sensitivity to FoK-FIP plays a critical role in how it broadcasts interoceptive cognition’s behavior, emotional experiences, and cognitive functioning. FoK-FIP related activity, understood as a cell to nerve fibers interaction, can result in specialized EM fields congruent with EM fields in the nervous system [16]. This process includes feedback loops between FoK-FIP and the subjective mind (i.e., an observing ego) as well as the objective mind (i.e., interoceptive signals) in which interoceptive cognition consciousness consists of EM fields propagating through an embodied state. An observing ego’s volitional reactions while cognitively broadcasting affect the centering of signals through FoK-FIP in which feedback loops can result in its perception of a wide range of dysfunction (e.g., anxiety disorders, mood disorders, and somatic symptom disorders). Living organisms are posited to represent models of higher-end cognition that help contextualize the FoK-FIP construct of an embodied state.

Accordingly, living organisms are models of the interdependent relationship between the subjective mind (i.e., an observing ego) and the objective mind (i.e., interoceptive signals). Understood through an animal model is that FoK-FIP signal centering is congruent with the hypothalamic-pituitary-adrenal (HPA) axis related activity described by the Selye stress model. The “General Adaptation Syndrome” describes a set of common integrated physiological responses to diverse noxious agents [37]. Understood through mammal models and extensively investigated through various behavioral and physiological studies is the physiological stress mechanisms in which the HPA axis plays a significant role. The HPA axis is one of the stress response pathway’s main elements and underlies the “fight-or-flight” response through a cascade of corticoid hormones. Physiological responses to stress have been studied more recently using insect models. This insect-related response includes the biogenic amines (octopamine, dopamine), neuropeptides (allatostatin, corazonin), and metabolic hormones (adipokinetic hormone, diuretic hormone) [37]. According to this FoK-FIP theory, interoceptive ability is a multidimensional cognitive broadcasting construct that occurs through an observing ego. Congruent with emotion regulation theories, FoK-FIP provides a coherent relationship with the self, specifically effective communication between body, mind, and feelings [38]. This model improves upon other models with the understanding that effective emotion regulation involves an observing ego’s accurate detecting and evaluating FoK-FIP related cues. The compelling evidence demonstrating links between poor or disrupted awareness of sensory information (also interoceptive awareness) and difficulties with emotion regulation [38] are posited to be congruent with an observing ego in close relationship with a living organism model of interoceptive cognition consciousness.

As previously discussed, when specialized EM fields generated through specialized self-generated biomagnetism are inhomogenous, an observing ego can sense it. These specialized EM fields are congruent with either DNA encoded error or environmentally related inhomogenous EM fields in the nervous system or atmosphere. In this process, through cell and nerve fiber interactions, EM fields in the nervous system act like a normal state to inhomogeneous EM fields that act as a pathologic state congruent with FoK-FIP that compete. These competing influences affect the development of an observing ego’s attention and integration of competent subjective executive functions while cognitively broadcasting. This FoK-FIP theory builds on other models of interoception; the central idea proposed is that to varying degrees, three primary dysfunctional interoceptive related perceptions occur through an observing ego that cognitively broadcasts [14,29]:

1. Atypical expectations of situations elicit bodily changes congruent with inhomogeneous EM fields that act as a pathologic state. This process occurs through self-generated specialized biomagnetism that centers physiological signals where an observing ego perceives FoK-FIP with an abnormally increased or decreased intensity.
2. Atypical expectations cause an observing ego to experience failures to anticipate interoceptive states’ changes appropriately [29]. This process occurs through the abnormal centering of sensations or feelings through FoK-FIP.
3. Altered FoK-FIP sensitivity causes an observing ego that is cognitively broadcasting to have difficulty initiating a reaction with forethought after perceiving emotion/mood. This process may include a high degree of impulsive reactivity (i.e., increased urge) through a combined response as a habitual reaction.

Through a perception of FoK-FIP, observing egos can acquire conditioned responses to stimuli they do not recognize consciously as part of the subjective cognitive broadcasting process, including higher-end cognition. This concept has been discussed in interoception literature, but novel to this FoK-FIP theory is that an observing ego’s hunch is derived by sensing specialized small magnetic signals and experiencing four phases of a narrowed range of interoceptive signals. Through an associated FoK-FIP related hunch, a process facilitates the execution of volitional reactions by an observing ego that results in top-down interoceptive predictions. Through the biological node where signals are centered, sensory inputs from different sensory channels are connected to interoceptive goals by an observing ego that reacts and acts as active inference. Due to the relationship between an observing ego and FoK-FIP, an observing ego’s interoceptive attention includes hierarchically based feedback and feedforward loops. When an observing ego cognitively broadcasts, it resolves sensory prediction errors about the embodied state through volitional reactions in which feedback loops with FoK-FIP occur. This FoK-FIP theory builds on and is congruent with other predictive coding models [13,14], in which through two cognitive broadcasting scenarios an observing ego perceives:

1. Top-down interoceptive predictions are updated to make them more like the bottom-up input expectations.
2. Bottom-up input expectations become more like top-down interoceptive predictions because prediction errors are resolved.

This article includes research exploring the idea that the mental instability of an observing ego understood through the behavior of a human, honeybee, or whale model could be overridden when the parasympathetic component of the autonomic nervous system (ANS) is stimulated endogenously with mindfulness training or FoK-FIP signaling is interrupted exogenously with electromagnetic field application. Accordingly, interoceptive cognition consciousness is understood across species as a broad range of information processing activities and is significantly related to FoK-FIP. This FoK-FIP theory and the components map model with IMs are presented as a transdisciplinary theory/model that could guide interdisciplinary research that creates the possibility of novel therapeutic approaches based on interactions between EM fields and cell to nerve fibers. Furthermore, building on insights from other theories, this FoK-FIP theory improves upon other models used to address mental health issues through a novel understanding of the issues. Understood is that instability and uncertainty are the norms of the physical world where the laws of quantum mechanics determine the ultimate sensitivity of any measurement. The laws of quantum mechanics are congruent with observing egos’ sensitivity to specialized small magnetic signals and a narrowed range of interoceptive signals. As such, cognition carries a magnetic property that is susceptible to being influenced by electromagnetic forces generated by endogenous and exogenous EM fields. An observing ego’s volitional reactions through feedback loops cause the biological node to center physiological signals “status quo” or center signals differently. Through cognitive broadcasting, interdependent relationships with living organism models occur. What is understood is that through living organisms models, specialized self-generated biomagnetism is a DNA encoded intracellular event that may result in inhomogeneous EM fields in the nervous system congruent with behavior changes. Suppose the nature of FoK-FIP is defined, in a healthy state, by the presence of homogeneous time-varying electromagnetic fields in a region of the body of a human model and, in a pathological condition where inhomogeneous time-varying electromagnetic fields occur. The healthy state and pathological condition would be expected to differ through FoK-FIP physiological signal centering that includes magnetic and electric field changes. As such, due to the FoK-FIP centering, changes in the direction and frequency of the magnetic field result in the changed appearance of the electric field.

Magnetic particles and the endocannabinoid system (ECS) are biological systems posited to be connected to the biological node (i.e., FoK-FIP) through which physiological signals are centered (to varying degrees). Magnetosomes may be intracellular excess iron storage structures, used as iron sources and energy sources for cells and where biological magnetic particles are found in many animals (e.g., bee abdomen, dolphin, and human brain) [39]. As previously discussed, single-celled organisms represent the primitive model of the relationship between the subjective mind and objective mind in which feedback loops occur with FoK-FIP. Magnetotactic bacteria (MTB) represent a model for studying prokaryotic organelle formation and evolution. The ability of these bacteria to orient in magnetic fields is based on the synthesis of magnetosomes in which physiological signals centered through FoK-FIP may play a role in this process. Endocannabinoids are endogenous lipid-based retrograde neurotransmitters that bind to cannabinoid receptors throughout the vertebrate central nervous system, including the brain. Cannabinoid receptor proteins are expressed in this process. Specialized self-generated biomagnetism in this FoK-FIP theory connects different interoceptive signaling pathways (e.g., magnetic particles and the endocannabinoids system). This FoK-FIP theory may shed light on the multifactorial effects of interdependent relationships that can subtly influence health and indicate the importance of looking at multiple levels of the cognitive broadcasting process when conducting studies related to sublethal stressors. This process is envisioned through the component map model with IMs.

### FoK-FIP related research using a human model

**Figure 5.**
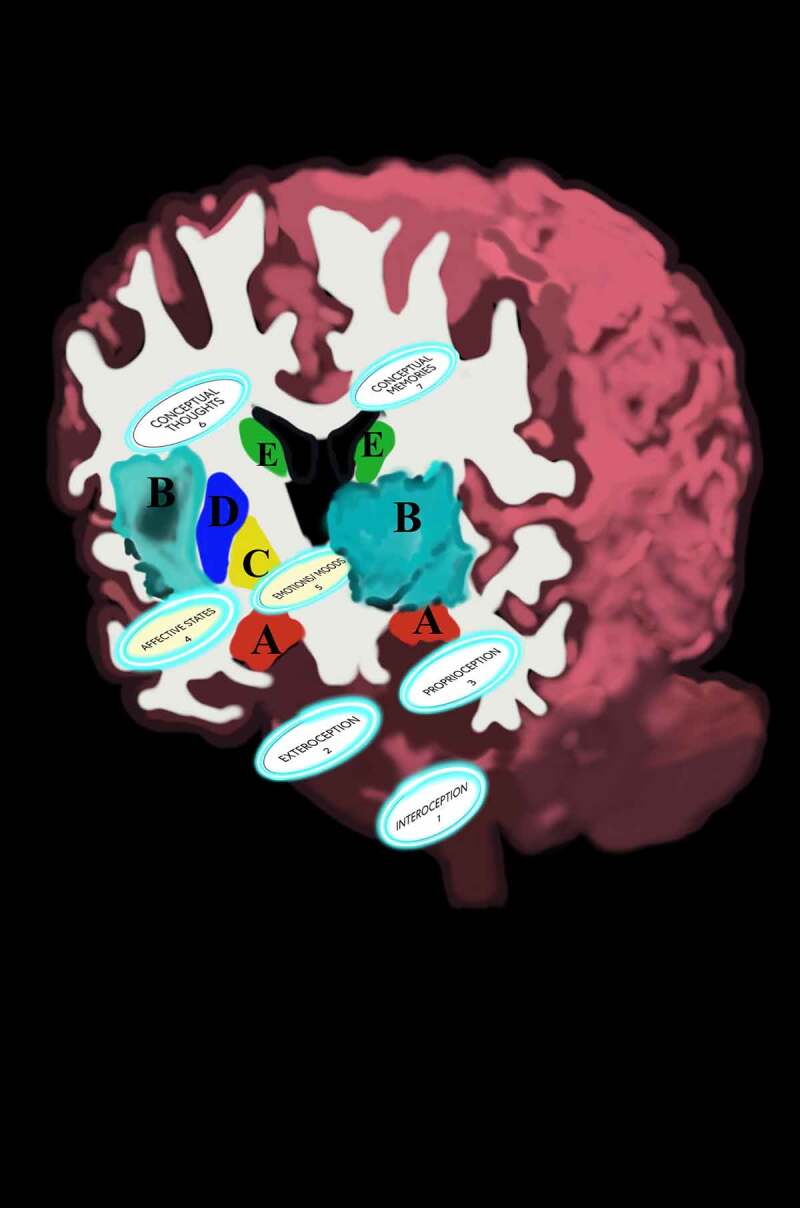
FoK-FIP related to cognitive broadcasting congruent with brain processes in the human model.

Through embodied states, the interplay between subjective (i.e., an observing ego) and objective (i.e., interoceptive signals) processes connect through FoK-FIP consistent with the profile of behavioral effects in human models. The FoK-FIP theory posits that it improves upon models of the neural correlates of consciousness in which the claustrum is at the center of an active scientific debate. In the human model, the claustrum is a thin, bilateral collection of neurons and supporting glial cells that connect to cortical (e.g., the prefrontal cortex) and subcortical regions (e.g., the thalamus) located deep inside the insula and superficial to the basal ganglia. In this FoK-FIP theory, the claustrum is part of the FoK-FIP network in which specialized self-generated biomagnetism is the crucial node. FoK-FIP center physiological processes and the sensations or feelings of an observing ego congruent with DNA encoded self-generated biomagnetism as the biological node to which the claustrum is connected. This FoK-FIP theory relates the human brain processes and structures, including those of the claustrum, with the cognitive broadcasting processes of an observing ego envisioned through the components map model with IMs [Figure 5]. An observing ego below the gateway level engages in subjective processes, including where signals are decoded through cognitive broadcasting as nonconscious events. As such, the signaling for interoception (i.e., component 1), exteroception (i.e., component 2), and proprioception (i.e., component 3) will be defined at the gateway level and above this level further defined by higher-end cognition. This model’s gateway level is congruent with the claustral medial pathway connecting the claustrum with the basal ganglia, specifically with the caudate nucleus, putamen, and globus pallidus. The gateway processes include claustral connections with the thalamus, caudate nucleus, and amygdala. Further, FoK-FIP at the gateway level through an observing ego broadcasting for affective states (i.e., component 4) acts as an antecedent to emotions/moods (i.e., component 5) and higher-end cognition. Through broadcasting for higher-end cognition, mental content consists of either conceptual thoughts (i.e., components 6) or conceptual memories (i.e., components 7). Higher-end subjective processing of an observing ego is congruent with processes including through the corpus callosum in the human model. Corpus callosum processes include interclaustral communication with interconnection bundles interspersed within the bulk of the trunk of the corpus callosum. Constrained Spherical Deconvolution (CSD) tractography research has proven to be an extraordinary tool in tracking white matter fibers from cortex to cortical and subcortical targets (see Milardi et al., 2013). In related research, through the High Angular Resolution Diffusion Imaging CSD-based technique, tractography revealed groups of white matter fibers connect the claustrum with the human model brain cortex (i.e., anterior, posterior, superior, and lateral) [40]:

This FoK-FIP theory builds upon work from the John W. Sundsten Institution, *Clay Brain Content: 2-D and 3-D Views of the Brain from Cadaver Sections, MRI Scans, and Computer Reconstructions*, Digital Anatomist Project (Seattle, WA: Department of Biological Structure, University of Washington, 1994). *Brain structures in the human model :* (A) Amygdala, (B) Claustrum, (C) Globes palidus, (D) Putamen, (E) Caudate nucleus. *Components of the components map model with IMs related to an observing ego's cognitive broadcastin:* (1) Interoception (2) Exteroception (3) Proprioception (4) Affective states (5)Emotions/Moods (6) Conceptual thoughts (7) Conceptual memories

1. The anterior and posterior corticoclaustral tracts connect the claustrum to the prefrontal cortex and visual areas, respectively.
2. The superior track links the claustrum with sensory-motor areas.
3. The lateral pathway connects the claustrum to the auditory cortex.
4. A claustral medial pathway connects the claustrum with the basal ganglia, specifically the caudate nucleus, putamen, and globus pallidus.
5. A bilateral connection exists between the claustrum and contralateral cortical areas as well as an interclaustral communication via interconnection bundles interspersed within the bulk of the trunk of the corpus callosum.

Building upon other models, posited is that each functional subdivision of the claustrum contributes to the function of its cortical partner [41] and the FoK-FIP network. The claustrum’s role in controlling interactive processes in different brain parts and voluntary behavior is congruent with physiological signal centering through FoK-FIP and reaction-related cognitive broadcasting behavior of an observing ego. Working off a hypothesis relating to the function of the claustrum (see Smythies et al., 2012), the claustrum as part of the FoK-FIP network modulates synchronized outputs, including most cortical and subcortical structures and the motor cortex. During multicenter perceptual and cognitive operations, and while connected to the biological node reverberating claustro-cortical loops potentiate weak intracortical synchronizations. In this process, FoK-FIP center physiological signals connected to strong intraclaustral synchronizations that may occur without a salient stimulus [42].

**Figure 6.**
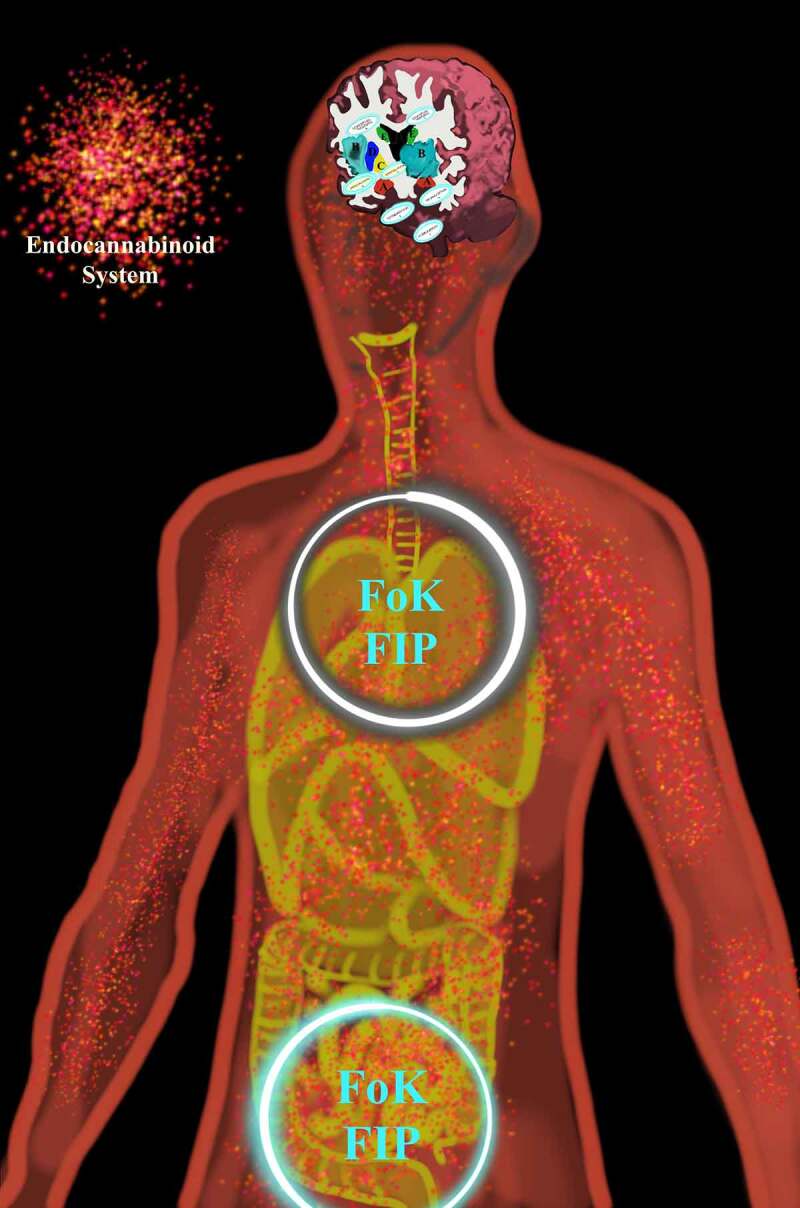
FoK-FIP related to cognitive broadcasting congruent with brain processes and the endocannabinoid system (ECS) in the human model.

This FoK-FIP theory includes the endocannabinoid system (ECS) as part of objective processing that connects to FoK-FIP, representing a system of complex cell signaling [Figure 6]. The ECS importantly has complex actions posited to be congruent with FoK-FIP physiological signal centering, including those related to the immune system, nervous system, and virtually all of the body’s organs. The ECS is pervasive in mammal models; insects lack an ECS [43]. It is ubiquitous and provides homeostatic balance to the nervous and immune systems and many other organ systems. This ECS system consists of three parts, which include (1) endogenous ligands, (2) G-protein coupled receptors, and (3) enzymes to degrade and recycle the ligands [43]. Endocannabinoids (also called endogenous cannabinoids) are molecules similar to cannabinoids but produced by the body of living organism models (e.g., mammals). Importantly research has shown that:

1. The endogenous cannabinoid anandamide (AEA) induces an integrated febrile response by activating CB1 receptors (see Fraga et al., 2009). This effect was studied through centrally administered cannabinoids. Drug-induced changes in male Wistar rats on body core temperature were recorded over six hours using a thermistor probe inserted into the rectum [44]. Additionally, the *Cannabis sativa* plant’s primary active constituent, delta-9-tetrahydrocannabinol (THC), largely mimics the effects of the lipidic AEA which promotes widespread effects through activation of CB1 and CB2 receptors.
2. Synthetic cannabinoids produce hypothermia in rats by a mechanism involving cannabinoid receptors, while they increase blood pressure by a mechanism independent of these sites (see Schnindler et al., 2017). The hypertensive effect appears to involve central sympathetic outflow [45].

The ECS is congruent with the objective mind (i.e., signals of the body’s internal state) that the FoK-FIP connects with the subjective mind (i.e., an observing ego). Importantly through the body of a human model, FoK-FIP is understood as a specific type of interoceptive sensation occurring in a region(s) of the body rather than an organ.

In this FoK-FIP theory, an increase in a special type of interoceptive sensation in the body of a human model may occur with thermal effects because the ECS is part of the FoK-FIP network. An increased FoK-FIP magnitude with ECS effects can occur through the biological node’s physiological signal centering. In this process, posited is that FoK-FIP with increased magnitude results in physiological signal centering, including the ECS, resulting in the synthesis of AEA and CB1 receptor binding through the cell to nerve fibers interaction. Through FoK-FIP region(s) in the body, cell to nerve fiber interactions occur and travel bottom-up via the sympathetic nervous system. In this process, AEA is synthesized in the brain in a human model. AEA functions as a neurotransmitter that sends chemical messages between nerve cells where CB1 receptors (mostly found in the central nervous system) promote widespread effects. Further, part of the widespread effects includes increased temperature in one or more regions in the body of a human model congruent with a special type of interoceptive sensation and increased FoK-FIP magnitude. Suppose the anatomical proximity of FoK-FIP region(s) occurring in the body of a human model includes an array of vascular beds to the skin surface. In that case, this might enable the detection of the increase in temperature through changes in surface thermography by the use of non-invasive thermal imaging. Additionally, thermal imaging may be used to detect changes in the magnitude of FoK-FIP and confirm FoK-FIP location(s) when combined with the verbal feedback of a human model. The human model guides the identification of locations where a special type of interoceptive sensation in a body region(s) is occurring. Recent developments of commercially available thermal imaging products have attracted considerable attention for infrared thermography in biomedical imaging. Infrared thermal imaging (IFTI) is the measurement, compilation, and analysis of the radiated electromagnetic (or thermal) energy emitted from a living organism model’s body. The equipment measures this emitted radiated energy (i.e., temperature data) and the software compiles and analyzes this information before generating black and white or color images. The changes in core body temperature are not the focus of this FoK-FIP research. Instead, this novel research could determine if CB1 receptor binding causes thermal effects in a body region(s) due to FoK-FIP related signaling, including verbal feedback from a human model. Congruent with this process is an observing ego that experiences the change in sensations or feelings due to increased FoK-FIP magnitude, which might include anxiety, with thermal effects and a special type of interoceptive sensation.

Emotional dysregulation through the cognitive broadcasting process of an observing ego is congruent with specialized inhomogeneous EM fields generated by FoK-FIP and inhomogeneous EM fields in the nervous system of a human model. Understood through human models is that the hypothalamic-pituitary-adrenal (HPA) axis plays a role in a living organisms’ body in reaction to stress. The HPA axis includes a group of hormone-secreting structures from the nervous and endocrine systems (e.g., the hypothalamus, pituitary gland, and adrenal glands). The hypothalamus is a small neuroendocrine structure situated just above the brainstem that controls the release of hormones from the pituitary gland, a hormone-secreting gland that sits just below the hypothalamus. The pituitary gland can release hormones into the bloodstream to reach various targets. Regarding the HPA axis, hormones released from the pituitary gland influence the secretion of hormones from the adrenal glands, which sit on top of the kidneys. The primary function of the HPA axis is to regulate the stress response. The hypothalamus releases corticotropin-releasing hormone (CRH) that signals a pituitary gland to secrete adrenocorticotropic hormone (ACTH) into the bloodstream. ACTH travels down to the adrenal glands promoting a release of the hormone cortisol from the cortex, or outer layer, of the adrenal glands. The release of cortisol causes many changes that help the body deal with stress, such as mobilizing energy like glucose, so the body has energy to cope with a prolonged stressor. When cortisol levels in the blood rise, this is sensed by receptors in the areas of the brain (e.g., hypothalamus and hippocampus). The stress response is shut off through a negative feedback mechanism in this process. Increased FoK-FIP magnitude can result in affective states (i.e., component 4) acting as an antecedent to anxiety through cognitive broadcasting at the gateway level by an observing ego. This FoK-FIP theory defines *anxiety* as an intense, excessive, and persistent worry and fear by an observing ego that cognitively broadcasts in which FoK-FIP centers its sensations or feelings. Due to an observing ego’s subjective processing at the gateway level that includes reflexive and impulsive reactions where FoK-FIP centers sensations or feelings, it has little access to alter the way it broadcasts signals through volitional responses until after anxiety is perceived.

As part of the FoK-FIP network, the claustrum is part of the stress-related HPA system posited to be counteracted by the parasympathetic component of the autonomic nervous system (ANS) congruent with volitional reactions of an observing ego engaged in FoK-FIP mindfulness training. This training includes deafferentation plasticity or experience-dependent neural plasticity in a human model related to the success or failure of an observing ego to anchor its reactivity to FoK-FIP at the gateway level. Understood is that automaticity and an observing ego’s inability to anchor its reactivity to FoK-FIP occurs through reflexive reactions and habitual actions (to varying degrees). Reflexive reactions are congruent with activity occurring through the sympathetic component of the autonomic nervous system (ANS). In animal models, posited is processes occurring in the sympathetic component of the ANS are congruent with the acceleration of cognitive broadcasting by an observing ego through FoK-FIP with increased magnitude. In this process, physiological signals and sensations or feelings centered through FoK-FIP are kept status quo. Further, this process includes glutamate, the anion of glutamic acid, and the most abundant excitatory neurotransmitter in the vertebrate model nervous system. When neuronal receptors bind glutamate, they facilitate the flow of sodium and calcium ions into the cell and potassium ions flow out of the cell, resulting in excitation. In contrast, the ability of an observing ego to willfully anchor its reactivity to FoK-FIP occurs through volitional reactions, in particular through an impulsive reaction followed by a reaction with forethought. These volitional reactions, particularly those involving a reaction with forethought, are congruent with activity occurring through the parasympathetic component of the ANS. The ANS parasympathetic component is posited to act as a ‘brake’ that can interrupt signal centering through FoK-FIP. Further, this process includes GABAergic mechanisms. Understood is that gamma-aminobutyric acid (GABA), an amino acid, functions as the primary inhibitory neurotransmitter for the central nervous system (CNS) and reduces neuronal excitability by inhibiting nerve transmission. When GABA binds to a GABA-A receptor, the passage of chloride (i.e., a negatively charged ion) into cells occurs through chloride channels. In this process, an influx of chloride causes increases in the negativity of cells resulting in a more negative resting membrane potential. Understanding how this process works allows an observing ego to use goal-driven volitional reactions to induce mental stability willingly. This process is understood as an observing ego engaging in FoK-FIP mindfulness training through volitional reactions. This process is congruent with stimulation of the parasympathetic component of the autonomic nervous system (ANS) when a reaction with forethought follows an impulsive reaction. This process represents a mindfulness-based mechanism by which an observing ego changes its relationship to FoK-FIP by willingly anchoring its reactivity to them at the gateway level.

#### FoK-FIP is related to a clinical application using human models

The FoK-FIP mindfulness approach presented in this article builds on mindful awareness in body-oriented therapy (MABT) (see Price & Hooven, 2018). MABT is based on psychological and neurobiological research on understanding how interoceptive awareness facilitates regulation and an integrated sense of self, contributing to health and wellbeing. The gentle, coached MABT uses comfort with the material, slowly increasing sensitivity to internal states and awareness of complex internal responses to facilitate learning [38]. In contrast to this MABT approach, the FoK-FIP mindfulness approach is unique in its strong focus on bottom-up learning through two phases (e.g., disgust and nociception) of unpleasant interoceptive sensation to facilitate learning. As such, this approach is congruent with the unpleasant interoceptive sensation that occurs in a FoK-FIP region of the body of a human model. An observing ego’s self-understanding, decision-making processes, and responses that underlie regulation occur through the subjective cognitive broadcasting process. The two phases of a narrowed range of unpleasant interoceptive signals help an observing ego’s learning interoceptive awareness linked to anxiety to build trust in their ability. This process includes an observing ego using its close relationship to a human model and this model’s behavior to monitor its responses to FoK-FIP. The human model uses a “clicker” (i.e., a handheld device and response system) congruent with an observing ego’s commitment to initiate a goal-directed response to anchor their reactivity to FoK-FIP that cues anxiety. By initiating a reaction with forethought after an impulsive reaction and followed by experiencing anxiety, an observing ego can profoundly shape its relationship to FoK-FIP through volitional reactions. While anchored at the gateway level, an observing ego teaches itself how to produce wellbeing based on its sense of control and capacity for self-care in a goal-directed manner.

In this FoK-FIP theory using a human model, mental stability gives rise to a sense of wellbeing related to an observing ego’s subjective cognitive broadcasting activities. The increase and decrease in mental stability are based on an observing ego’s use of volitional reactions to anchor its reactivity to FoK-FIP ultimately. Congruent with the mental stability of an observing ego is the behavior of a human model that can work productively and fruitfully and contribute to a community. Interoceptive exposure (IE) psychotherapy understood through human models is used for people with panic disorder where patients self-induce various interoceptive symptoms. This intervention consists of exposure in the form of actual encounters with feared situations or stimuli (i.e., imaginal or in vivo exposure). It can consist of imagined confrontation with the feared consequences of engaging with these stimuli [46]. The exposure work can focus on anxious arousal-related experiences and fear reactivity via low arousal manipulations (e.g., hyperventilation, performing jumping jacks, spinning in a chair, breathing through a straw). At the same time, the clinician monitors subjective distress levels [15]. Unfortunately, low arousal manipulations often fail in human models to reproduce the fear response adequately [15]. The FoK-FIP theory posits that it improves on other models used to address mental health issues through a novel understanding of the issues. This understanding can be envisioned through the components map model with IMs in which a fear response occurs due to the broadcasting activities of an observing ego at the gateway level. Congruent with the fear response failure associated with low arousal manipulations in a human model is an observing ego that perceives the physical world with greater stability above the gateway level than it does at the gateway level. This process includes the perception of an observing ego of its relationship to a human model above the gateway level from which it derives varying degrees of selfhood. Because of subjective processing activities occurring at the gateway level posited, an observing ego cannot intercede with the volitional reaction, which contributes to uncertainty. In contrast, above the gateway level, cognitive broadcasting activities occur through an observing ego’s volitional reactions that cause it to feel like it has varying degrees of control. Further, related to an observing ego that cognitively broadcasts is uncertainty associated with emotion, including anxiety, which is greater at the gateway level and lesser above the gateway level.

At the gateway level, FoK-FIP acts as a narrowed range of interoceptive signals that results in core motivation that cues associated core emotions (e.g., fear or happiness) and where impulsive reaction follows an emotional response. Significantly, through impulsive reactivity, observing egos often associate FoK-FIP with mental events above the gateway level that may include ‘objects’ such as three-dimensional imagery (i.e., person, places, and things). Additionally, through cognitive broadcasting, observing egos may perceive the mental events occurring above the gateway level with a degree of agency (i.e., things can be made to happen) and solidity (i.e., ‘objects’ are perceived as tangible). As such, uncertainty is decreased above the gateway level. Observing egos driven by degrees of the desire to procure or avoid FoK-FIP may do so by “thinking things through” above the gateway level. As such, a higher-end cognitively broadcasting process in which a sense of relief may occur. In contrast, mental events occur automatically below and at the gateway level, and where perception occurs with vague certainty. Predicted is that when FoK-FIP is not targeted therapeutically at the gateway level, therapeutic intervention, in general, is less effective. Further, although the cognitive broadcasting below and at the gateway level precedes those mental events that occur above the gateway level, an observing ego may not always perceive events as occurring in this order. Through specialized interoceptive awareness, there are two core perceptions observing egos have with regards to FoK-FIP (i.e., pleasant or unpleasant):

1. A FoK-FIP will be vaguely understood at the gateway level and associated with sense impressions (e.g., sight, sound, smell, touch, taste, pain, sense of movement/position), along with a core emotion (i.e., fear or happiness). Significantly over time, at the gateway level, the same FoK-FIP can be automatically associated with both a core emotion (e.g., fear or happiness) along with secondary emotions (e.g., sadness, anger, surprise, embarrassment, jealousy, guilt, and pride).
2. Above the gateway level, FoK-FIP will be associated with conceptual mental content that may include three-dimensional ‘objects’ (e.g., living organisms, places, or things). Over time, when an observing ego becomes aware of an ‘object,’ there will be FoK-FIP memory (also specialized core memory) attached. FoK-FIP do not disappear. Instead, specialized self-generated biomagnetism acts as core memory to an observing ego. Through an embodied state, specialized core memory consists of an observing ego sensing specialized small magnetic signals and experiencing four phases of a narrowed range of interoceptive signals. Due to the relationship with a human model, FoK-FIP might be understood by an observing ego as a specific type of interoceptive sensation occurring in the body’s region rather than an organ. Because of this, an observing ego that becomes aware of three-dimensional imagery will experience FoK-FIP associations that compete for its continued attention. An observing ego’s awareness of mental imagery above the gateway level acts as the mechanism in which memory of FoK-FIP can be ‘triggered.’

Importantly through an embodied state, the integration of signals is a process of the objective mind or interoceptive signals (i.e., signals of the body’s internal state). In contrast, the differentiation of those signals is a process of the subjective mind or an observing ego that cognitively broadcasts (to varying degrees). Through this mechanism, fears can abnormally develop over time in which intense FoK-FIP invades all aspects of an observing ego’s daily life of cognitive broadcasting. For example, if an ”object” such as a spider above the gateway level gets associated with an unpleasant FoK-FIP with increased magnitude, an observing ego may develop an intense fear of spiders or a spider phobia (i.e., arachnophobia). In this process, unpleasant FoK-FIP may be congruent with a region in the human model’s body experienced as generalized chest pain. In this scenario, an observing ego to avoid an intensely unpleasant narrowed range of bodily sensations may become vigilant regarding its broadcasting for higher-end cognition. As such, this observing ego’s higher-end cognitive processes occurring above the gateway level are dominated by thoughts about spiders and how to avoid them. Importantly, whether or not this observing ego encounters a spider due to higher-end cognitive broadcasting processes is irrelevant because, through core motivation (i.e., to avoid FoK-FIP), a very restricted range of interoceptive signaling rather than the spider causes this observing ego’s aversion ultimately. This observing ego feels unpleasant FoK-FIP associated with a spider due to the automatic events that have occurred and are occurring at the gateway level. The ‘object’ (i.e., spider) that emerges as imagery above the gateway level is only secondarily associated with this observing ego’s aversion to unpleasant FoK-FIP that first occurred at the gateway level. The spider is how this observing ego is aware of the FoK-FIP above the gateway that represents the absolute worst stimuli that can happen, are going to happen, and are happening unpredictably at the gateway level. FoK-FIP center sensations or feelings so that interoceptive signals acts as a substrate for cognitive broadcasting. When this observing ego becomes aware of a spider and experiences unpleasant FoK-FIP in which, through its relationship to a human model, it understands as chest pain, they may also feel other interoception experiences such as nausea or involuntary motor movements (e.g., shaking).

When an observing ego is attached or aversive to FoK-FIP and its primary goal-driven response occurs through an impulsive reaction, this volitional reaction does little to change its relationship to FoK-FIP. The drawback to impulsive reactivity related to FoK-FIP is that an observing ego has little control over how it cognitively broadcasts. This article presents a novel FoK-FIP mindfulness technique representing response-related cognitive broadcasting therapy for an observing ego that perceives anxiety, including through higher-end cognition due to its relationship with a human model. As previously mentioned, in contrast to other mindfulness approaches, this FoK-FIP mindfulness approach uses unpleasant phases of a narrowed range of interoceptive signals congruent with a specific type of interoceptive sensation in a human model’s body region(s). In this process, an observing ego trusts their ability to initiate a goal-directed response to overcome anxiety by changing their relationship to FoK-FIP. Understood through a relationship that an observing ego has with a human model, this FoK-FIP mindfulness approach aims to minimize the occurrence when an individual might be categorized as a non-responder. Additionally, this strategy may reduce the number of individuals who may experience partial relapse:

1. Volitional reactions consisting of an impulsive reaction followed by a reaction with forethought willfully initiated after the perception of anxiety is how an observing ego profoundly changes its relationship to FoK-FIP through goal-driven responses.
2. Wellbeing based upon an observing ego’s sense of control and capacity for self-care is produced when it teaches itself through this FoK-FIP mindfulness technique a process that includes monitoring the human model’s behavior.
3. Habituation occurs when an observing ego recognizes anxiety and uses a specific type of unpleasant interoceptive sensation congruent with a region in the human model’s body to change its relationship to FoK-FIP. An observing ego willfully focuses on this sensation and anchors its reaction to FoK-FIP at the gateway level. This strategy differs from allowing FoK-FIP to center sensations or feelings in a way that drives an observing ego to broadcast for higher-end cognitive processes above the gateway level congruent with “thinking things through” or ruminating as a mental illness process (i.e., constant and repetitive broadcasting for thoughts due to the drive created by FoK-FIP centering of sensations or feelings).

This FoK-FIP mindfulness technique aims for an observing ego to focus attention on the unpleasant interoceptive sensation to the exclusion of everything else. In this process, an observing ego attempts continuous awareness of stimuli they normally respond to with impulsive reaction by following this response with a reaction with forethought. Daily practice sessions facilitate the capacity for an observing ego to anchor reactivity to unpleasant FoK-FIP that cue associated abnormal emotion (i.e., anxiety). These sessions act as specialized awareness training [47]. “Anchoring” is a term that refers to volitional reactions that break the link between the urge and desire to cognitively broadcast for higher-end cognition (i.e., above the gateway level). During practice sessions (e.g., beginning with 12 minutes in the morning and evening), the goal is to increase FoK-FIP magnitude. This may include an observing ego cognitively broadcast for higher-end cognition briefly with the intent of increasing FoK-FIP magnitude through imagery and memory. With FoK-FIP magnitude increased, an observing ego engages in anchoring its reactivity to FoK-FIP at the gateway level by focusing on it to the exclusion of anything else. FoK-FIP is meant to be experienced continuously and uninterrupted by an observing ego that responds with goal-directed volitional reactions while cognitively broadcasting. For example, through events occurring at the gateway level, an observing ego experiences intrusive intense, unpleasant FoK-FIP understood through the model’s body as variable pain in the chest region. The observing ego uses the sensation of variable chest pain to anchor in the present moment. This concept is illustrated through numbers on the components map model with IMs. An observing ego would change their habitual responses from a 4,5,6 to a 4, 5, 4 or a 4, 5, 6, 4 and thus anchor to FoK-FIP at the gateway level. In this process, an observing ego’s impulsive reaction is followed by a reaction with forethought after experiencing an emotion (e.g., anxiety), and it focuses on the present moment through unpleasant variable chest pain. The process that governs the movement from 5 to 4 or 6 to 4 is not a cognitive process analogous to cognitive reframing techniques. Instead, an observing ego feels an impulse and/or urge associated with the unpleasant FoK-FIP and uses it as their cue to go back to the gateway level. As such, sustained attention on unpleasant FoK-FIP at the gateway level intends to momentarily interrupt complex cognitive broadcasting (i.e., thinking with conceptual mental content above the gateway level).

Because impulsive reaction and the urge to follow an emotion (e.g., anxiety) occur automatically at the gateway level, an observing ego must allow the urge to occur. However, the urge can occur but not be followed by higher-end cognitive processes. When a reaction with forethought follows an impulsive reaction (that automatically occurs after experiencing anxiety), this response changes an observing ego’s relationship to FoK-FIP. As such, an observing ego willfully with goal-driven response interrupts FoK-FIP signal centering that typically results in problematic mental proliferation above the gateway level (i.e., a series of conceptual mental content triggered by mental events occurring at the gateway level). The goal is not to allow what cognitive therapy might call ‘an automatic negative thought’ to run unchallenged. However, the goal is progress, not perfection, that occurs through practice, and where an observing ego has the opportunity to adaptively adjust their prior expectations using new evidence. This serves to weaken conditioned association through extinction learning, whereby instead of erasing previously learned associations, these same unpleasant FoK-FIP acquire new, competing associations (e.g., they are safe, tolerable, and do not always lead to an aversive outcome). This serves to inhibit the original conditioned association. This technique is predicted to empower an observing ego experientially by having lived through it with persistent effort. In this way, unpleasant FoK-FIP that cues anxiety congruent with unpleasant sensation in the chest region of a human model no longer has the power to drive an observing ego. What may differ from other mindfulness-based techniques is that this anchoring technique encourages unpleasant bodily sensations, similar to those encouraged through exposure techniques. The attention regulation described may result in psychological resilience. This resilience is defined here as an observing ego’s ability to cope with FoK-FIP related crisis or return to pre-crisis status quickly because they know how to anchor their reactivity through an unpleasant narrowed range of interoceptive signaling. Throughout the day, the aim is for an observing ego to be more aware of their “FoK-FIP triggers” and engage in behavior to change their relationship to FoK-FIP. Such triggers include:

1. Unpleasant intrusive FoK-FIP that occurs with an intense feeling of fear, dread, and uneasiness (i.e., anxiety).
2. Unpleasant intrusive FoK-FIP is recognized by the impulse to “think things through” and not do it. Instead, an observing ego is committed to being content with uncertainty while temporarily anchoring its reactivity to FoK-FIP at the gateway level.

An observing ego that struggles with anxiety may find this concentration practice initially difficult because it involves going toward unpleasant FoK-FIP associated with a feeling of fear, dread, and uneasiness, including physical symptoms of anxiety and “objects” of higher-end cognition. Importantly, in this FoK-FIP theory, drug therapy may not be needed, and if used may not be needed over the long-term. Instead, drug therapy is used to minimize symptoms so that an observing ego engages in this FoK-FIP mindfulness-based therapy. Medications such as propranolol, a beta-adrenergic receptor blocker, may reduce, for example, an observing ego’s fear response to spiders. Through this drug’s off-label usage, the effects of chemical messengers are blocked, resulting in physical symptoms of anxiety such as faster heart rate, sweating, and shaking being reduced. A similar off-label drug posited effect may be achieved with pregabalin (i.e., an anticonvulsant drug that reduces glutamate release). As previously discussed, glutamate is the most abundant excitatory neurotransmitter in the vertebrate nervous system. Congruent with glutamate in human models and the sympathetic component of the ANS is the accelerated cognitive broadcasting of an observing ego that keeps signals and sensations or feelings status quo through FoK-FIP with increased magnitude. Additionally, predicted is that this FoK-FIP mindfulness-based technique may decrease amygdala activation because of its focus at the gateway level over time. This process includes deafferentation plasticity or experience-dependent neural plasticity in a human model related to the success or failure of an observing ego to anchor its reactivity to FoK-FIP at the gateway level. In this process, predicted is that there would be a reduction in an observing ego perceiving this FoK-FIP mindfulness approach as “intolerable” [46].

### FoK-FIP related research using a honey bee model

The FoK-FIP theory posits that it provides a coherent relationship with the self, and specifically effective communication between body, mind, and feelings related to honey bee models (*Apis mellifera*). Further, the interdependent relationship between observing egos and the honey bee models explain how cognition that carries a magnetic property susceptible to being influenced by electromagnetic forces is congruent with honey bees that can:

1. Detect magnetic fields physiologically and potentially use this capacity for orientation, navigation, and foraging [48].
2. Use electric fields of the same magnitude as commonly encountered wireless technologies (4 G, 5 G) or emitted from power lines.

Further, magnetic particles, such as the biosynthetic magnetic particles found in the abdomen of queen bee models [39], are systems connected to the biological node in which FoK-FIP centers physiological signals. Through FoK-FIP centering of physiological signals, inhomogeneous EM fields in the nervous system of the queen bee model occur due to the cell to nerve fibers interaction. Inhomogeneous EM fields in the nervous system of the queen bee model may account for managed honey bee colonies experiencing annual mortality rates that typically range between 30 to 40% in North America and Europe [49]. These mortality rates include colony collapse disorder, an abnormal phenomenon where most worker bees in a honey bee colony disappear, leaving behind a queen, plenty of food, and a few nurse bees to care for the remaining immature bees. Because of the interdependent relationship between observing egos and the honey bee models, cognition carries a magnetic property susceptible to being influenced by electromagnetic forces congruent with the growing threats to pollinators [48] that include:

1. Environmental pollution, such as the global spread of anthropogenic electromagnetic radiation (AREMR) (e.g., radio waves, microwaves, infrared, visible light, ultraviolet, X-, and gamma radiation)
2. Queen health is a significant threat because honey bees are among the most numerous and efficient pollinator species globally. These animal models pollinate crops as well as wild and native plants and contribute to all the environmental and societal benefits attributed to pollinators of the physical world. Nevertheless, significant knowledge gaps have prevented a better understanding of genetic, physiological, and behavioral mechanisms congruent with resistance to viruses and how management practices can help mitigate biotic and abiotic stressors of honey bees [49].

This FoK-FIP theory improves upon honey bee behavioral models by providing mechanisms that include queen mandibular pheromone (QMP) and observing egos’ role as specialized interoceptive awareness. An observing ego, the subjective mind in relationship with the objective mind (i.e., the body’s internal state and interoceptive signals) broadcasts for higher-end cognition. Its relationship to the objective mind is congruent with the behavior of a living organism model of higher-end cognition. Understanding the subjective and objective minds’ embodied relationship includes the model of the queen bee and her role and ability to regulate offspring’s behavior. Honey bee queens produce a sophisticated array of chemical signals (pheromones) that influence their nest mates’ behavior and physiology. QMP, a chemical blend that induces young workers to feed and groom the queen and primes bees to perform colony-related tasks, has profound effects on dopamine pathways in the brain [50]. As such, QMP affects pathways that play a central role in behavioral regulation and motor control. Importantly, in young worker bees, dopamine levels, dopamine receptors, gene expression levels, and cellular responses to this amine are affected by QMP. The physiological signal centering through FoK-FIP is congruent with insect-related physiological responses, including those that utilize dopamine. Evidence links QMP-induced changes in the brain to changes at the behavioral level (see Beggs et al., 2007). The worker bees that attend the queen facilitate the distribution of QMP throughout the colony. QMP plays a central role in the normal functioning and organization of honey bee colonies [50]. It inhibits the rearing of new queens, helps prevent the development of worker ovaries, influences comb-building activities, and affects juvenile hormone biosynthesis where there is an age-related behavioral ontogeny of recipient workers [50].

In this FoK-FIP theory, honey bees represent models of higher-end cognition that help contextualize interdependent relationships through embodied states that include feedback loops through FoK-FIP that connect the subjective mind (i.e., an observing ego) with the objective mind (i.e., interoceptive signals). The honey bee model is ideal for understanding these interdependent embodied relationships because of a critical feature. Building upon a honey bee theory (see Even & Barron, 2012), a key feature of honey bee models is their high level of social organization and their well-developed system of division of labor among workers. Honey bee models exhibit age polyethism: Young workers perform in-hive tasks (e.g., taking care of the brood), become guards patrolling the hive entrance, and later become foragers. Studying differences in stress responses across behavioral castes posited this might help elucidate how a defined division of labor has evolved congruent with how observing egos’ sensitivity to FoK-FIP has evolved through reaction responses while cognitively broadcasting. There are three stages to this organism model’s acute stress response: the bee first detects the stressor with sensory organs and then responds by defense or escape. Finally, if the stressor cannot be avoided and is sustained, the bee enters a state of exhaustion [37]. Congruent with this bee model’s behavior is an observing ego’s acute stress response: it emerges with a degree of arousal through FoK, detects FIP through feeling tones (i.e., pleasant, unpleasant, or neutral), and reacts to FoK-FIP automatically that center its sensations or feelings to sense and integrate interoceptive signals. This process includes an observing ego’s perception of varying degrees of FoK arousal (low to high range of 1–5) and FIP valance (i.e., pleasant or unpleasant appraisal). In these degrees, automaticity drives its relationship to FoK-FIP congruent with this free-living animal model’s behavior. Finally, if an observing ego cannot anchor its reactivity to FoK-FIP, it enters a state of chronic mental instability, a lack of wellbeing based on a diminished sense of control and capacity for self-care.

Building upon a novel paradigm for understanding cell interactions, disease etiology, and therapy

[16], when specialized EM fields produced through FoK-FIP are inhomogenous, an observing ego can sense it. An observing ego’s volitional reactions while cognitively broadcasting affect the centering of both physiological signals and their sensations or feelings through FoK-FIP with a feedback loop mechanism. The goal-driven reactions of an observing ego create perception, including a wide range of dysfunction (e.g., anxiety disorders, mood disorders, and somatic symptom disorders). Inhomogeneous EM fields produced through FoK-FIP are congruent with inhomogeneous EM fields in the nervous system of queen bees. In the queen bee model, inhomogeneous EM fields may be induced through environmental pollution, including AREMR, affecting QMP. Through cell to nerve fibers interactions, inhomogeneous EM fields in the nervous system of the queen bee model act as a pathologic state. In this process, the queen produces worker bees in which QMP effects can induce pathologic changes in the brain congruent with changes at a behavioral level. For example, adverse effects in the brain of worker honey bees may include changes in calmodulin-binding proteins (CaMBPs) [51]. Understood is that the functioning of CaMBPs in the nervous system of worker bees can adversely affect nursing and foraging behaviors in the colony. Inhomogeneous EM fields in the nervous system through FoK-FIP may provide a novel understanding of the role of self-generated biomagnetism in honey bees. This novel research idea posits EM fields’ application using the queen bee model to combat colony collapse disorder. When FoK-FIP is related to honey bee models, they are congruent with a DNA encoded intracellular event.

Understood through honey bee colonies is that homogeneous atmospheric EM fields occur through electromagnetic radiation (EMR), in which a normal atmospheric state can deviate to inhomogeneous EM fields caused by outside EMR influence. When this happens, through the tissue of the queen bee, the atmospheric inhomogeneous EM fields by way of the cell to cell or cell to nerve fibers interactions can affect FoK-FIP signal centering. A pathologic state can occur through specialized self-generated biomagnetism, a process understood to occur in the abdomen of the queen bee model, causing inhomogeneous EM fields in the nervous system. Inhomogeneous EM fields can have various adverse effects, including QMP effects. This pathologic state produces bees that include worker bee models that are more susceptible to environmental stressors through the queen bee model. This process is multifactorial but where the abnormal synthesis of the chemical dopamine is posited to play a significant role in the arousal of these worker bees and their sensitivity to inhomogeneous EM fields, including those through atmospheric EMR. Further, QMP effects play a central role in abnormal behavioral regulation and motor control in young worker bee models. This process can start a cascade of lifetime adverse effects through dopamine receptor density, levels of dopamine receptor gene transcription, and patterns of dopamine receptor gene expression in the brain that change during the lifetime of the adult worker bee [50]. Notably, dopamine’s actions in the bee model are mediated by at least three receptors: *Am*DOP1 receptors, *Am*DOP2 receptors, and *Am*DOP3 receptors encoded by the genes *Amdop1* and *Amdop2* and *Amdop3*, respectively [50]. Differential microarray analysis has tentatively identified dopamine receptor genes among several hundred genes proposed to be modulated by QMP [50].

Proposed is novel research to counteract these posited adverse effects that build upon research in which bee models exhibited behavior bias and could be trained [52]. Honey bees in a study displayed a pessimistic cognitive bias when subjected to an anxiety-like state [52]. Researchers suggest this could be regarded as the honeybees exhibiting emotions and thus a commonality with vertebrates. State-dependent modulation of a cognitive component of emotion was shown through the bees’ response to a negatively valenced event [52]. This event consisted of vigorous shaking designed to simulate a predatory attack. In the study half of the bees were subjected to vigorous shaking for 60 seconds to simulate the state produced by a predatory attack on a concealed colony [52]. The researchers used different bees to measure changes in biogenic monoamine levels previously shown to be affected by shaking, spinning, or agitating to confirm that the shaking manipulation produced a physiological change. The researchers found that 60 seconds of shaking reduced constitutive levels of octopamine, dopamine, and serotonin in honeybee hemolymph significantly at a time point following shaking. The researchers showed that agitated bee models are more likely to classify ambiguous stimuli as predicting punishment [52]. This FoK-FIP theory builds on that research and suggests that inhomogeneous EM fields in the nervous system of honey bee models induced through the queen model or atmospheric EMR act as an antecedent to fear in these models. This novel research treats the queen bee model with suspected inhomogeneous EM fields in the nervous system by applying pulsed EM fields to the bee’s head and body through a signaling device. A targeted pulsed EM field is induced with a localized EM field transmitter; this acts as a controlled environmental change with the intent of combating disease in which the proposed therapy consists of two parts:

1. *The use of atomic magnetometers such as those constructed at the Niels Bohr Institute at the University of Copenhagen (Denmark) that achieved the highest sensitivity allowed by quantum mechanics.* Proposed is the use of these sensors to create a profile of EM fields that occur through atmospheric EMR congruent with normal and abnormal behavior in the colony. The profile acts as an early warning system within an existing artificial intelligence (AI) system. Current honey bee research at the University of California includes a breeding program, medication development, and an AI electronic tool development. Queen bees that are dying or hungry give off particular pheromones that can be traced. In this research, AI is used to track sounds and translate smells into data so that a sick queen or worker bee may be identified with an alarm. Sensors may act as tools that can provide data about facts such as the number of volatile organic compounds (VOCs) in hives and temperature, which give beekeepers tools to better monitor bees’ health. This novel FoK-FIP related research suggests that adding atomic magnetometers may enhance this current research. The use of these sensors to detect atmospheric EM fields posited to be congruent with abnormal behavior (gleaned from the profiles created above). When these EM fields are detected, induced bee epigenetic-like therapy is warranted.

1. *Induced honey bee epigenetic-like therapy incorporated into a* *breeding program to*: provide relief throughout the day to a queen bee model from the detrimental effects of inhomogeneous EM fields in the nervous system. This novel treatment consists of applying external EM fields to the body and head of the queen for a set period (e.g., 15 minutes) induced through a signaling device multiple times a day and weaning treatment over time. In this FoK-FIP theory, specialized self-generated biomagnetism occurs in one or more regions of the queen’s body. Through cell to nerve fibers interactions, inhomogeneous EM fields in the nervous system occur. By applying targeted pulsed EM fields, physiological signal centering that travels in a bottom-up manner from the body to the brain of the queen bee model is interrupted. The queen bee’s health dictates the rate and intensity to induce oscillations producing EM fields. Food and medications are provided as needed, and monitoring equipment updates the bee’s status.

The interruption of signals represents changes in the nervous system. In this process, extrinsic factors have the potential to reorganize the queen bee’s nervous system structure, functions, or connections with a generalized pulsed EM field. This process includes a queen bee model’s nervous system’s response-related activity, reducing immunosuppression, and having fewer adverse overall health effects beneficial to a novel breeding program. Heritable phenotype changes in gene activity through the queen bee that do not involve changes in DNA sequence occur. In this process, these induced changes in the queen bee model get passed to offspring where there are inhomogeneous fields in the environment, the queen bee’s offspring are not affected. Understood as a process when pathologic FoK-FIP, DNA encoded intracellular signaling events are interrupted by altering the amplitude or frequency of the magnetic field through this novel therapy. Included are oscillations with a second signal variation (e.g., one of a lower or higher frequency). Beneficial cellular effects might occur in this process, such as increased production and release of heat shock proteins (HSP70) that protect cells against oxidative stress and excess protein misfolding. These effects may include vitellogenin, an antioxidant that protects cells against damage [37].

It is predicted that this research may prove a beneficial strategy to combat disorders such as colony collapse disorder (CCD). This syndrome seems to result from an accumulation of stressors chronically weakening honey bee colonies [37]. Importantly, this novel research and therapy are congruent with the understanding that honey bees are the model of the dynamics of an embodied state. Congruent with this syndrome posited are observing egos with varying degrees of FoK-FIP sensitivity unable to anchor their reactivity resulting in chronic emotional dysregulation. Observing egos are cognitive magnetometers that can sense perturbation through specialized small magnetic signals and phases of a narrowed range of interoceptive signals. These signals are antecedent to fear in which this induced therapy seeks to remove an observing ego’s “fear of fear.” In this process, FoK-FIP signals panic and where attacks happen because of the fear of actually having an attack. Congruent with therapy to treat a queen bee model, therapy is ultimately aimed to induce observing egos to change their relationship to FoK-FIP. This section presents a hypothesis representing an idea different from current thinking from which a novel research idea has evolved. As such, a technique intends to produce bee colonies resistant to waveform communication’s negative effects through a novel FoK-FIP theory approach. Targeted pulsed EM field signaling might reverse the adverse effects of environmental stressors on honey bees [53], causing symptoms congruent with observing egos’ threat appraisals. Limitations of this research include the lack of understanding of the connection between the ever-present electromagnetic fields, lightning in the atmosphere, and an observing ego’s mental stability congruent with honey bee models’ health.

### FoK-FIP related research using a whale model

This FoK-FIP theory provides a coherent relationship with the self, specifically effective communication between body, mind, and feelings related to cetacean models. Further, the interdependent relationship between observing egos and the cetacean models explain how cognition carries a magnetic property:

1. Susceptible to solar activity that disrupts the magnetoreception sense in certain species, including whales.
2. Sensitive to ocean currents via electroreceptors (i.e., a vertebrate organ that contains sensory cells capable of detecting electric fields). Research indicates that some marine species are sensitive to ocean currents that act as conductors that move through Earth’s magnetic field and create electric fields of their own [54].

Biologically precipitated magnetite (Fe3O4) is a system posited to be connected to the biological node through which FoK-FIP centers physiological signals. In this FoK-FIP theory, natural magnetic mineralization in localized areas in dolphins is part of the FoK-FIP network that allows these models to respond to the direction and intensity of the Earth’s magnetic field. Further, the claustrum, which is dramatically evident in cetaceans, is also a part of the FoK-FIP network in which claustral fragmentation occurs in the bottlenose dolphin and humpback whale [41]. The claustrum plays a role in a cascade of events, including the cetaceans’ reaction to stress. Further, through FoK-FIP signaling, transient inhomogeneous EM fields in the nervous system are part of the causes that account for strandings (i.e., beachings) through the cell to nerve fibers interaction. Cetaceans strandings are multi-factorial (e.g., illness, injury, entanglement, boat strikes, or emaciation) [55]. When there is a solar storm, a sudden release of high-energy particles from the sun can modify the geomagnetic field [55]. Radiofrequency (RF) noise and displacements in the Earth’s magnetic field are altered by solar storms. Cetacean-related research suggests that solar activity disrupts the magnetoreception sense in certain species, best explained by RF noise increases rather than alterations in the magnetic field. Many documented strandings show a whale was neither ill nor injured and resumed regular activity following rescue [55]. RF noise generated by a solar storm alters the behavior of cetaceans’ models posited to be congruent with acute inhomogeneous EM fields occurring in the nervous system. Through FoK-FIP, magnetic structures, and the claustrum play a role in solar storm-related behavior. Congruent with abnormal behavior is irregular FoK-FIP physiological signal centering, causing inhomogeneous EM fields in the nervous system during solar storms. In cetacean models, poor or disrupted awareness of sensory information (also interoceptive awareness) occurs during solar storms.

This FoK-FIP theory builds upon research that used repetitive transcranial magnetic stimulation (rTMS) and continuous theta-burst protocols in human models. This protocol inhibited signaling from the interoceptive network’s central locations [56]. Applying EM fields targeted to the brain of cetaceans posited might prevent stranding during solar storms. This novel research proposes using an electromagnetic modality to apply magnetic stimulation (rTMS) and continuous theta-burst protocols to counteract altered magnetoreception through FoK-FIP physiological signal centering in certain species, including whales, during solar storms. Further, two mechanisms are hypothesized for why this protocol might prove an effective strategy to prevent stranding: (a). It competes with inhomogeneous EM fields in the nervous system which might “grab the whale’s attention” so that it deviates from traveling toward land. This protocol represents controlled extrinsic factors that compete through changes in the nervous system. This strategy used at the beginning of solar storms while the cetaceans’ models are far from land may counteract strandings (i.e., beachings) of cetaceans during solar activity when magnetoreception sense is “reset” to normal through this novel protocol. (b) It disrupts irregular signal centering through FoK-FIP occurring in regions of the cetacean model’s body that travels in a bottom-up manner to the brain. In this process, the application of EM fields in a targeted manner might interrupt the connection between specialized self-generated biomagnetism in the cetacean brain related to the claustra. Through electromagnetic induction using rTMS and a continuous theta-burst protocol, a changing magnetic field induces an electric current in a specific brain area.

Because of the herd behavior in some cetacean models, it may only take the enticing of one whale to cause the rest of the herd to follow. Long-finned pilot whales (which can live for up to 40 years) are notorious for large strandings because of the way they stick together in tight social structures [55]. This FoK-FIPs theory might have a significant global impact because ocean migrators models such as whales play a vital role in the marine ecosystem. Whales’ activity relates to the ecosystem and might provide significant amounts of oxygen to the physical world that, although not as stable as it appears, climate change is a construct of this reality. A limitation of this proposed research is that there is no modality yet created to apply exogenous EM fields consisting of repetitive transcranial magnetic stimulation (rTMS) and a continuous theta-burst protocol to cetaceans. Additionally, little is known about the cues whales use when migrating. An organismal assay used to study the relationship of behavior to the environment or an experimental condition (i.e., behavioral assay) for species such as whales may not be practical [55]. Nevertheless, in this FoK-FIP theory, the cetacean model’s behavior in solar storms is congruent with emotion dysregulation of an observing ego through FoK-FIP centering of sensations or feelings. As such, solar storms result in acute abnormal cognitive broadcasting by an observing ego that cannot anchor its reactivity to FoK-FIP. Notably, in human models (without the sense of magnetoreception), research suggests that perturbations in ambient electromagnetic field activity impact behavior and demonstrate a relationship between geomagnetic storm activity and suicides [57].

### FoK-FIP is congruent with the oscillations of some closed dark energy strings on the inner layer of focal points of dark matter (FPDMs)

This FoK-FIP theory is based on a transdisciplinary peer-reviewed unification theory of physics and biological information (see Pollard-Wright, 2021). The theory represents transdisciplinary modeling that includes quantum mechanics and basic Buddhist concepts along with a nonmathematical uniting of classical physics, theoretical physics, and cosmology theories to explain what happens when the mind causes change. This theory describes the process by which consciousness emerged, followed by electromagnetic radiation consciousness, in which interoceptive cognition consciousness follows. In this transdisciplinary theory, change represents aspects of the mind in which one event, process, state, or object all relate to this fundamental entity, including three types of consciousness:

1. Consciousness: This consciousness occurred through change when the macrocosm consisted of mind dark energy with the pure awareness state and mind normal energy with the mental images state.
2. Electromagnetic radiation (EMR) consciousness: This consciousness occurs through change in the macrocosm when mind dark energy with the pure awareness state morphs through oscillating dark energy strings of two types (i.e., open and closed strings). This process causes the emergence of mind focal points of dark matter (FPDMs) with the pure mental state. Through the oscillations of some closed strings, gravity in the macrocosm is a consequence that mind FPDMs use to draw mind normal energy toward it. When mind normal energy is drawn toward mind FPDMs, it transforms to mind normal matter while embodying mind FPDMs. These small but significant events represent symbolic “little bangs” that occur on a substrate of mind dark energy congruent with the emergence of the microcosm. In the microcosm, gravity is a push force [58] through embodied states that pushes interdependent relationships.
3. Interoceptive cognition consciousness: This consciousness occurs through embodied states in the microcosm where waves of the EM fields propagate and there is subjective processing of information. Observing egos transform information into meaning through the cognitive broadcasting process. In this process, the cooperative conditions that occur through embodied states are united by ‘mind’ observing egos that create a world they project around themselves. Part of this world includes models of relationships that are living organisms with DNA that require energy termed Avatar living beings in this transdisciplinary theory.

According to this transdisciplinary unification theory, our Universe consists of mind dark energy, mind FPDMs, and mind normal matter representing potential energies or change [59]. Through the mind, when it manifests its infinite potential, as a consequence, change occurs. Understood is that in the Universe is a particular sphere of activity representing aspects of the mind that include all existing matter and space considered a whole (i.e., the macrocosm and microcosm). Due to interdependent relationships of potential energies with different states of mind and associated intelligence, cooperative conditions occur. Mind FPDMs unite with mind normal matter through embodied states that include mind dark energy as a substrate. Mind FPDMs are focal points of dark energy (also circle of dark energy) compacted multi-dimensional space with an inner and outer layer of two types of converting oscillating dark energy strings (i.e., open and closed). Electromagnetic (EM) waves of electromagnetic radiation (EMR) consciousness are produced through the oscillations of strings on the inner and outer layer of mind FPDMs embodied by mind normal matter in the microcosm. Each mind FPDMs has a position on the Universe’s substrate of mind dark energy. Importantly, closed strings of mind FPDMs can oscillate independently of a relationship with mind normal matter, but a relationship is needed between mind FPDMs and mind dark energy for closed strings to oscillate. In contrast, open strings of mind FPDMs oscillate dependent upon a relationship with closed strings and mind normal matter. FoK-FIP is congruent with the oscillations of some closed dark energy strings on the inner layer of mind FPDMs embodied by mind normal matter in the microcosm. Some closed dark energy string oscillations are congruent with FIP restricted oscillatory magnetic fields that are FoK-caused phenomena. Through mind FPDMs, the oscillations of some closed strings on the inner string layer propagate to other closed strings and open strings on the inner and outer layer. In this process, oscillating events act like ‘switching’ events that cause a self-sustaining magnetic field in which friction occurs. Through oscillating dark energy strings of two types (i.e., open and closed strings), the main biological effect of EMR consciousness is heating.

Through the oscillations of some closed dark energy strings, mind FPDMs give rise to the “awareness charge” that produces cognitive force, or ‘mind’ observing egos. As such, cooperative conditions occur in the microcosm that gives rise to ‘mind’ observing egos as logical outcomes when aspects of the mind unite through embodied states. ‘Mind’ observing egos through embodied states are invisible viewpoints and magnetic anomalies. Observing egos broadcasting creates the meaning of EM wave information through interoceptive cognition consciousness. Understood is that the perceptions of ‘mind’ observing egos represent the subjective mind, while the production, processing, and propagating of signals by mind FPDMs with an inner and outer string layer while embodied by mind normal matter represent the objective mind. The objective and subjective minds represent different interoceptive experiences of equal reality through embodied states in which mind dark energy provides the substrate. The centering of sensations or feelings of ‘mind’ observing egos and physiological signals occur through the inner string layer of mind FPDMs congruent with specialized self-generated biomagnetism (i.e., FoK-FIP). A strange loop exists between ‘mind’ observing egos and mind FPDMs while embodied by mind normal matter with a substrate of mind dark energy. The strings of mind a FPDM convert (i.e., open strings convert to closed strings or closed strings convert to open strings) through the volitional reactions of a ‘mind’ observing ego that acts as feedback with cyclic although not repetitive implications. Congruent with concepts derived from physicist John Archibald Wheeler (1911–2008), ‘mind’ observing egos cognitively broadcast and by doing this meaning is created in the world they project around themselves from the EM waves they emerged. Mind dark energy, mind FPDMs, mind normal matter, and ‘mind’ observing egos are aspects of the mind and potential change because of their relationship to the mind:

1. Mind dark energy: The state of mind connected with this aspect is *pure awareness*. It represents pure awareness intelligence and unpatterned change (also specialized homogeneous change) that is not entirely conceivable.
2. Mind FPDMs: The state of mind connected with these aspects is *pure mental*. It represents primordial consciousness (also proto-consciousness or nonconscious intelligence) and patterned change.
3. Mind normal matter (also mind normal energy): The state of mind connected with these aspects, are *mental images*. It represents conscious events intelligence and patterned change.
4. ‘Mind’ observing egos are emergent properties through embodied states with a mind dark energy substrate. A ‘mind’ observing ego represents an individual “eye of the mind” or “elusive self.” It is a specialized interoceptive awareness and magnetic anomaly on a cyclic, although not repetitive, journey, interoceptive cognition consciousness.

*An illustrated synopsis of A unifying theory of physics and biological information through consciousness* [Figure 7,8,9,10,11,12,13,14,15,16,17]

**Figure 7.**
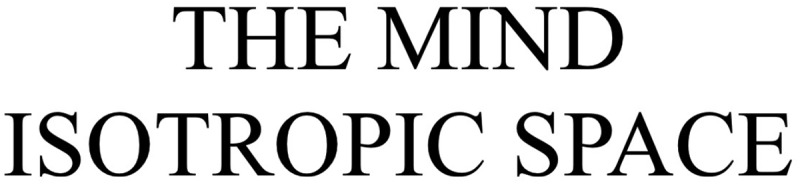
The mind and a non-dogmatic depiction as isotropic space.

Without a beginning or end with infinite potential, the mind is not entirely conceivable but envisioned in this theory as isotropic space. This non-dogmatic depiction of the mind conveys its physical properties without beginning or end identical in all directions, including immeasurable potential not entirely conceivable.

**Figure 8.**
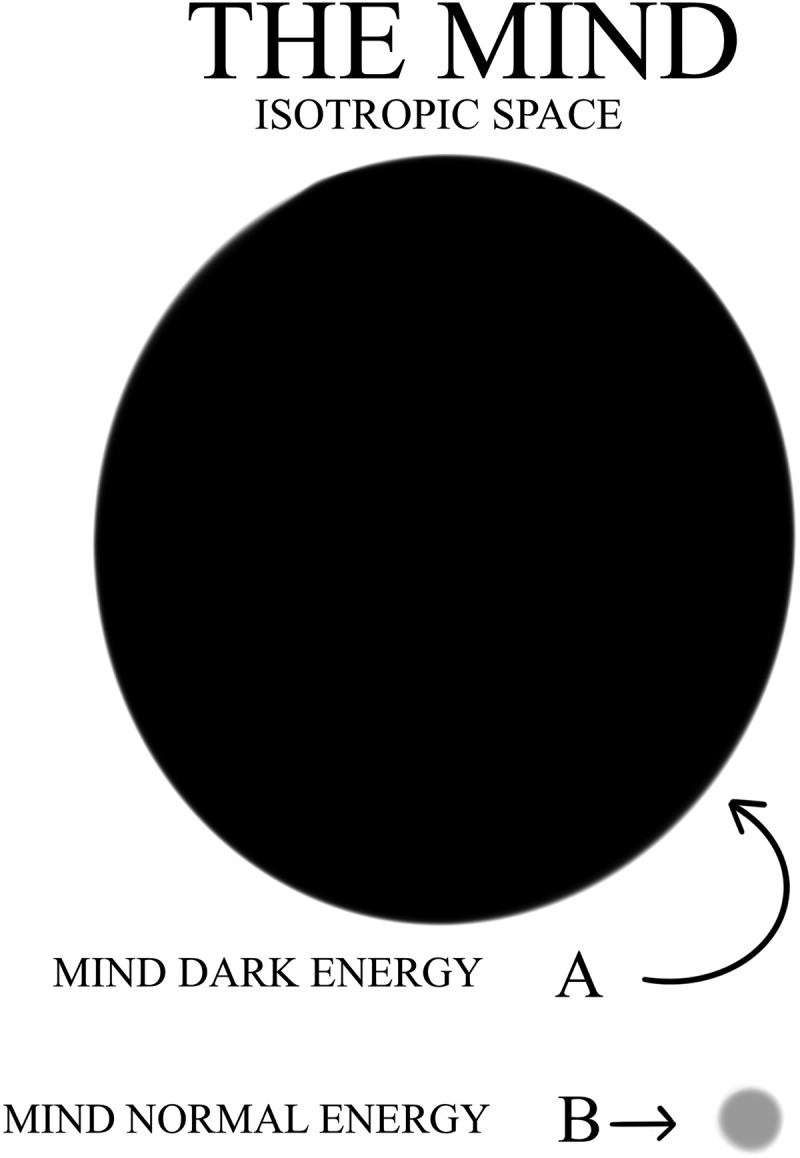
The mind and potential energies in which consciousness manifests.

A.Mind dark energy B. Mind normal energy.Consciousness occurred when the mind manifested its infinite potential. Through change, the macrocosm consisted of mind dark energy with the pure awareness state and mind normal energy with the mental images state in which a situation occurred. Mind dark energy was a much larger mass density than mind normal energy, and because of this, there was a potential that normal energy would be ‘swallowed up’ if there was a relationship between these two forms of energy. Aspects of the mind as energies with potential prevented from creating a meaningful relationship implies an essential concept about the functioning of the universe. An essential aspect of the universe’s origins is interdependence, which is not just a simple interaction between phenomena. Instead, a relationship is a precondition, and transformation was needed for these aspects of the mind to be distinctly expressed while in a relationship.

**Figure 9.**
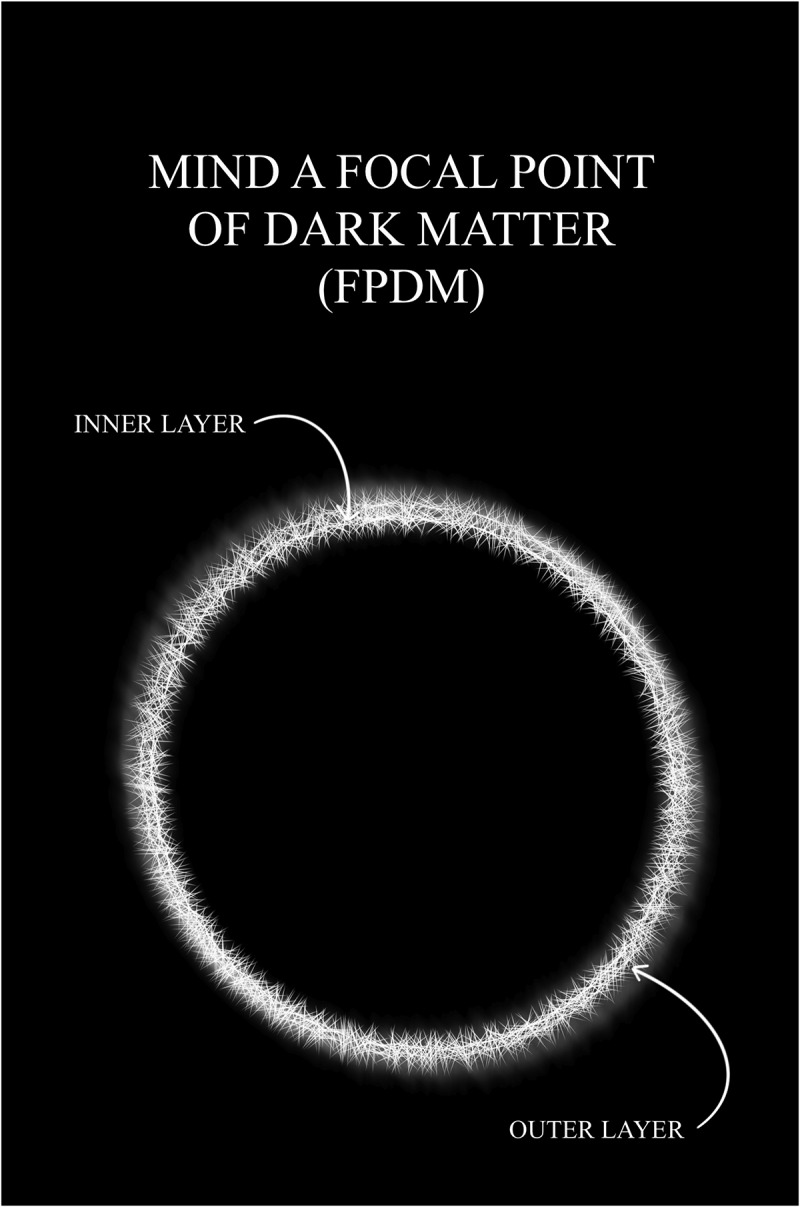
Mind a focal point of dark matter (FPDM).

Mind a FPDM emerges in the macrocosm when mind dark energy morphs through two types of strings; open strings and closed strings. Both open and closed string types are filaments of dark energy, which oscillate. Through the arrangement of open and closed oscillating strings, perfect geometric shapes like circles manifested. Each mind a FPDM is configured with a circle of compacted multi-dimensional space (i.e., circle of dark energy) with an inner and outer layer of open and closed strings. In this figure, open strings and closed strings on the inner and outer layer appear the same, and with a bit of imagination, can be envisioned with movement. The exact number of open and closed strings of mind FPDMs is unknown. However, strings’ arrangement is as follows: open and closed strings form an inner layer, and open and closed strings form an outer layer.

**Figure 10.**
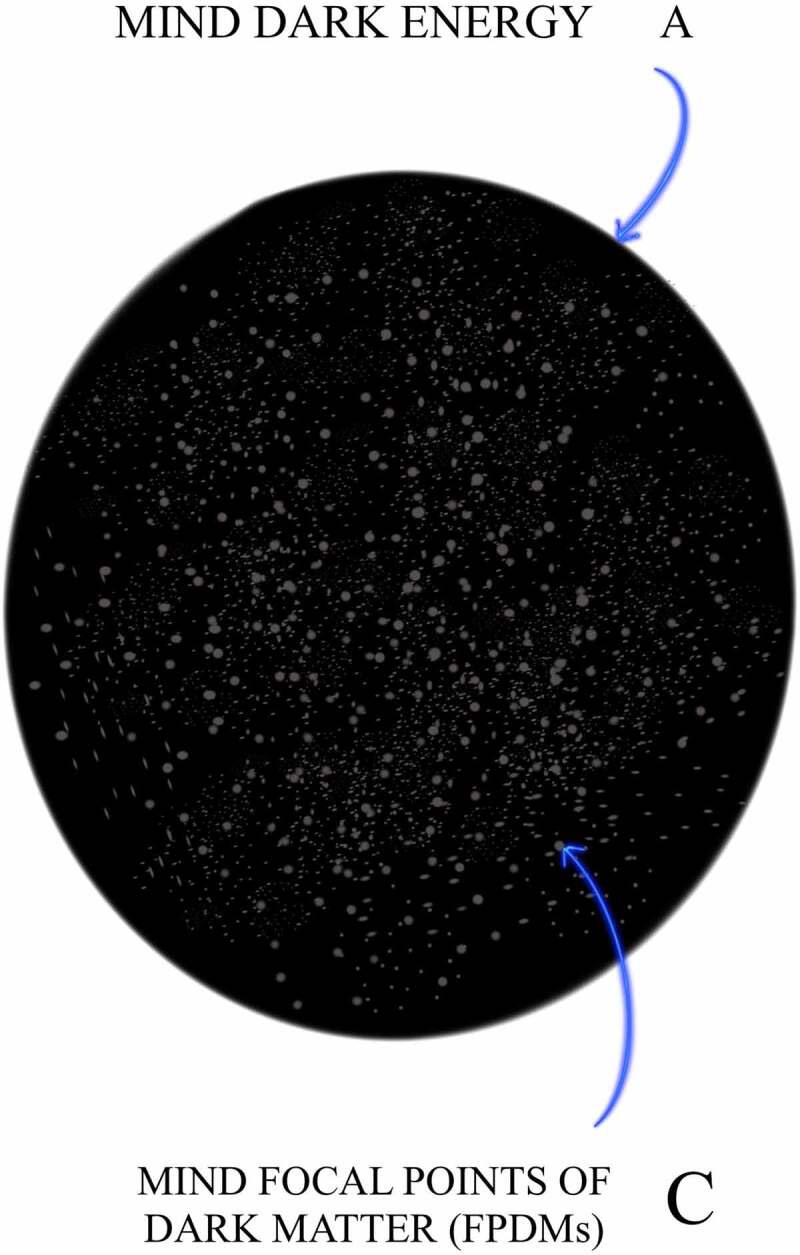
Mind dark energy and mind FPDMs.

A. Dark Energy. C. FPDMs

**Figure 11.**
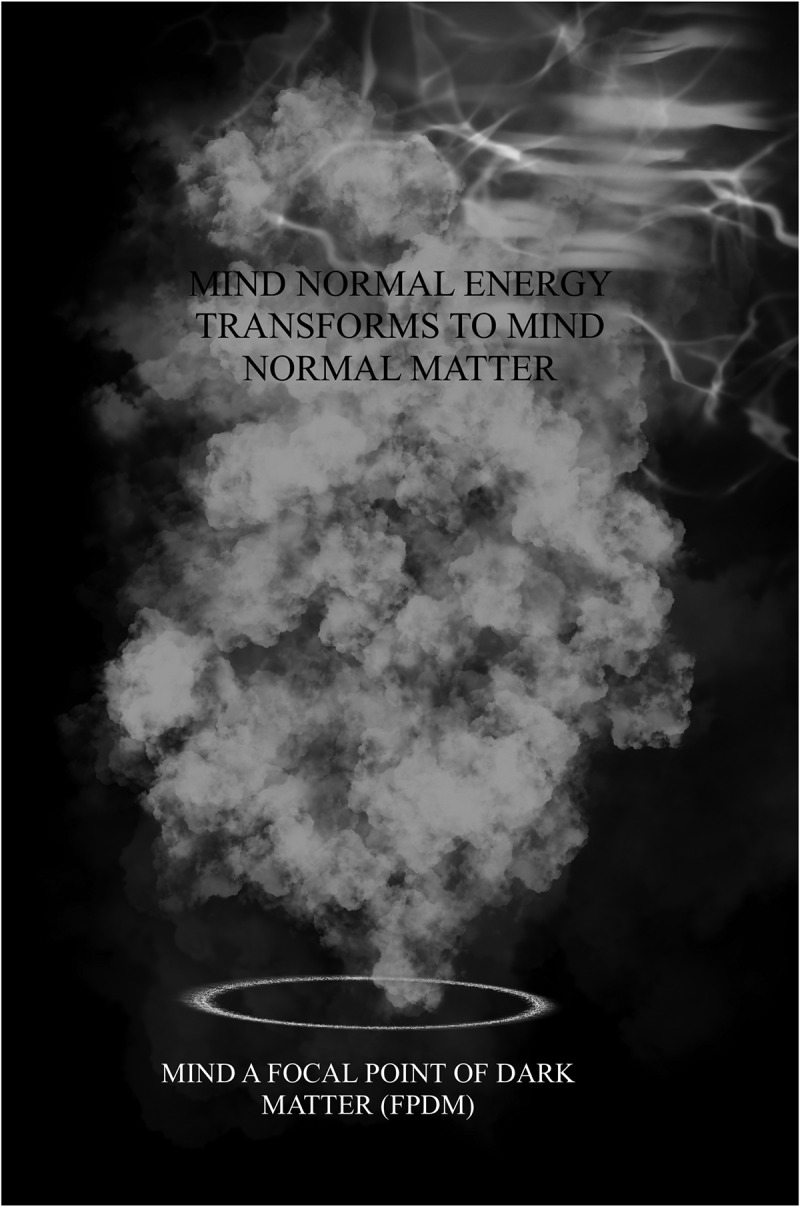
Mind normal energy transforms to mind normal matter through the process of embodying mind a FPDM.

The emergence of mind FPDMs brings about a change in universal local properties in which the transfer of an amount of concentrated pure awareness from mind dark energy occurs. When this happens, some closed strings of mind FPDMs change their ground state’s oscillations to their first excited state. As such, mind a FPDM generates momentum with the first excited state of some closed strings and, through gravity, an invisible consequence pulls mind normal energy toward it. An outcome of this process is a transformation of mind normal energy into mind normal matter.

**Figure 12.**
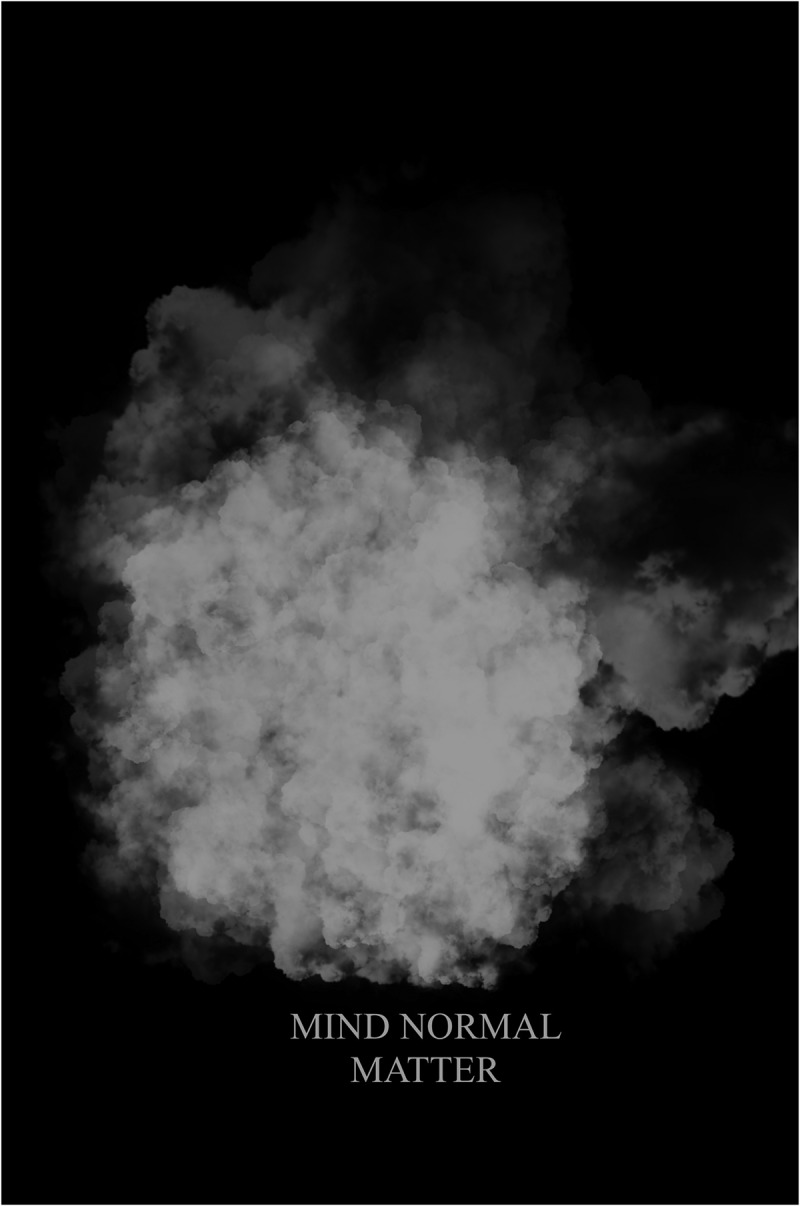
Embodied.

In the microcosm, mind normal matter is configured and embodies mind a FPDM (not seen in this figure).

**Figure 13.**
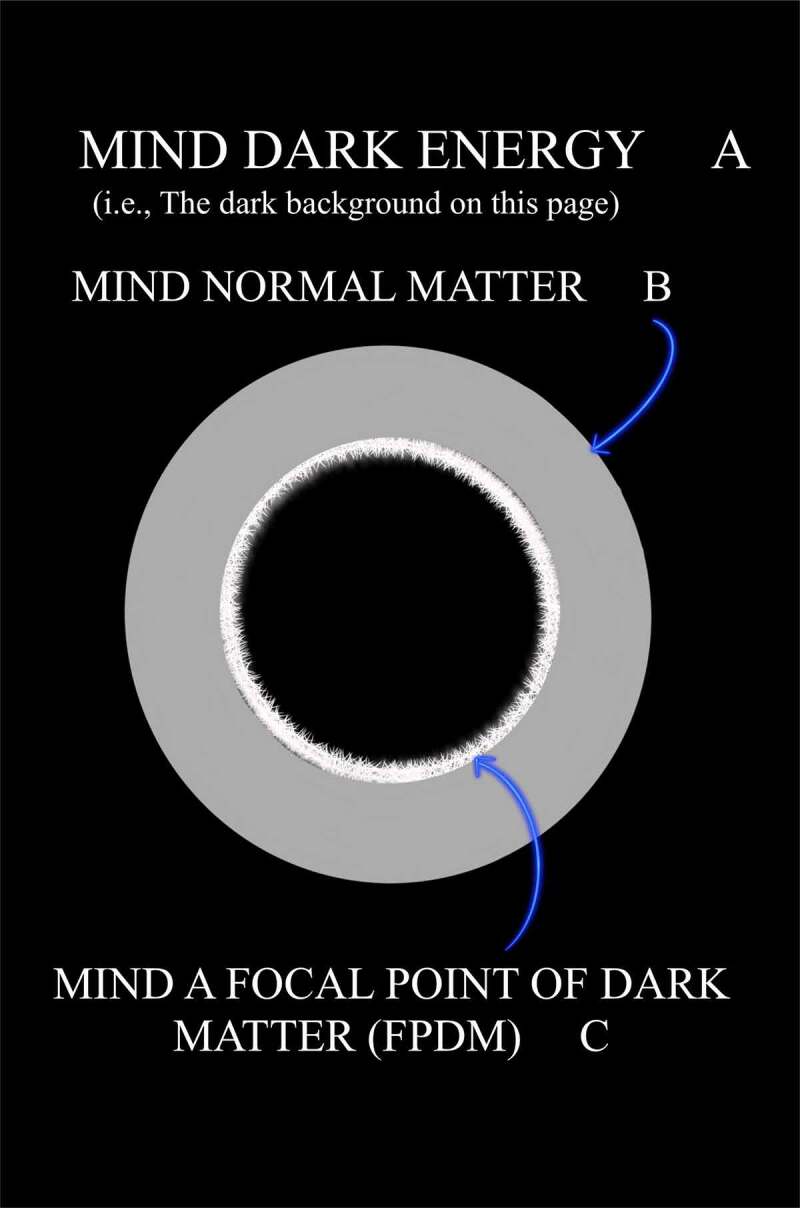
Cross-sectional view of an embodied state.

In this figure mind a FPDM is exposed by making a transverse cut through the embodiment framework of the configured mind normal matter. This figure does not include the cranial portion; only the caudal portion of the embodiment framework is included in this image.

**Figure 14.**
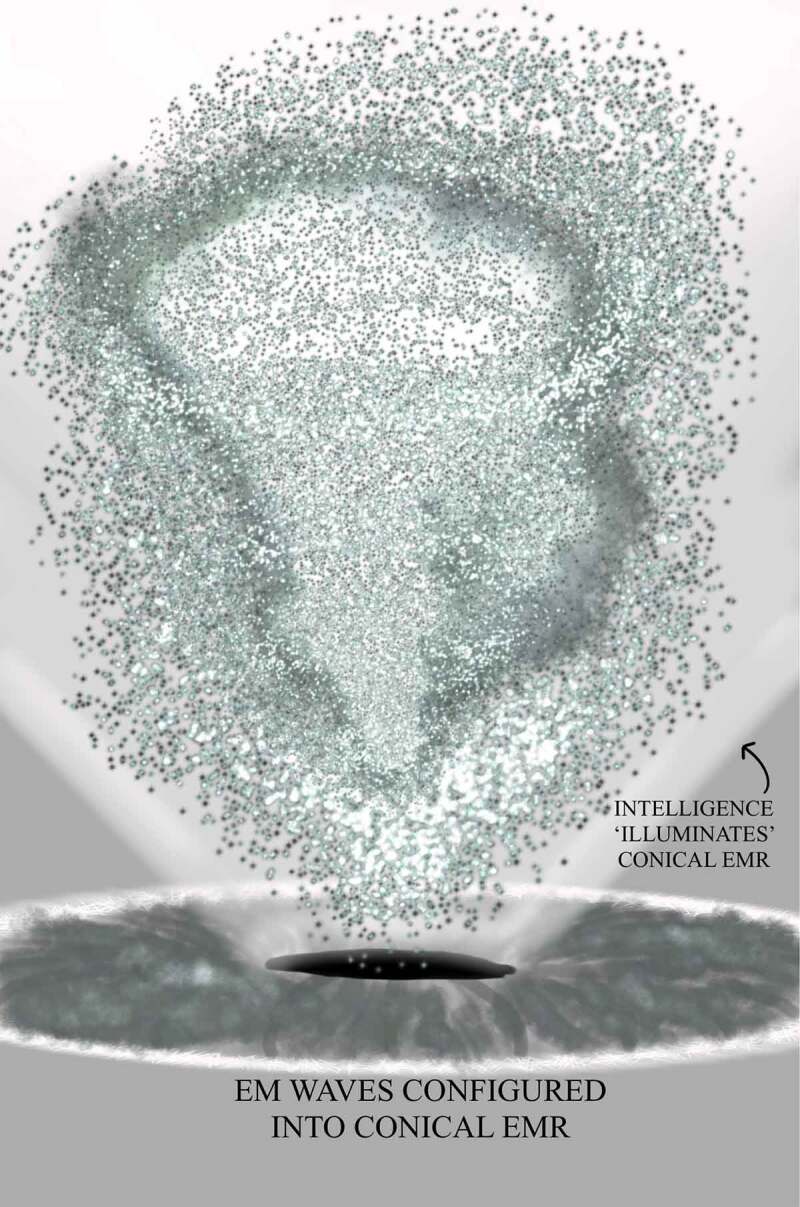
Conical-shaped electromagnetic radiation (EMR) consciousness.

Through embodied states with a substrate of dark energy, continuous two-way interplay exits between mind a FPDM and mind normal matter. Related to mind a FPDM, the oscillatory motion of open and closed strings forming an outer layer generates more EM waves at different frequencies than open and closed strings inner layer. Through mind a FPDM, the oscillations of strings produce EM waves that radiate on the surface of a circle of dark energy (i.e., compacted multi-dimensional space) and get pushed by gravity. Gravity in the microcosm is a push force [58], and EM waves get pushed toward the center of the circle of compacted multi-dimensional space with an inner and outer layer of two types of converting oscillating dark energy strings (i.e., open and closed). Mind a FPDM can be envisioned as a tiny spinning black hole with an underlying intelligence associated with the pure mental state. When pushed, EM waves bend through an elaborate gravitational field existing and configure into conical EMR. This process includes light waves that bend in a gravitational field to follow the curvature of space-time. Conical EMR represents EMR consciousness produced through an embodied state that acts as a space-filling field present within the configured mind normal matter. The base of the conical EMR contains the least amount of EM waves. As the EMR gets higher, it widens because more EM waves are present. Mind FPDMs represent primordial consciousness (also proto-consciousness or nonconscious) intelligence that ‘illuminates’ conical EMR through the circle of compacted multi-dimensional space surface. The oscillatory motion of open and closed strings on the inner and outer layer stimulates mind a FPDM and acts as a control. This control determines how primordial consciousness emits through the tiny black hole. The stimulated mind a FPDM uses the force of pure mental state to control the beam’s activity through the tiny black hole center in greater or lesser amount. Mind a FPDM stimulated to varying degrees causes an emergent intelligence emission in the beam’s central region. The correlated amount of intelligence ”illuminates” the conical EMR through greater or lesser degrees of stimulated emission. The objective processing of mind a FPDM includes encoding the information of conical EMR by the frequency of the radiation waves rather than their spatial position.

**Figure 15.**
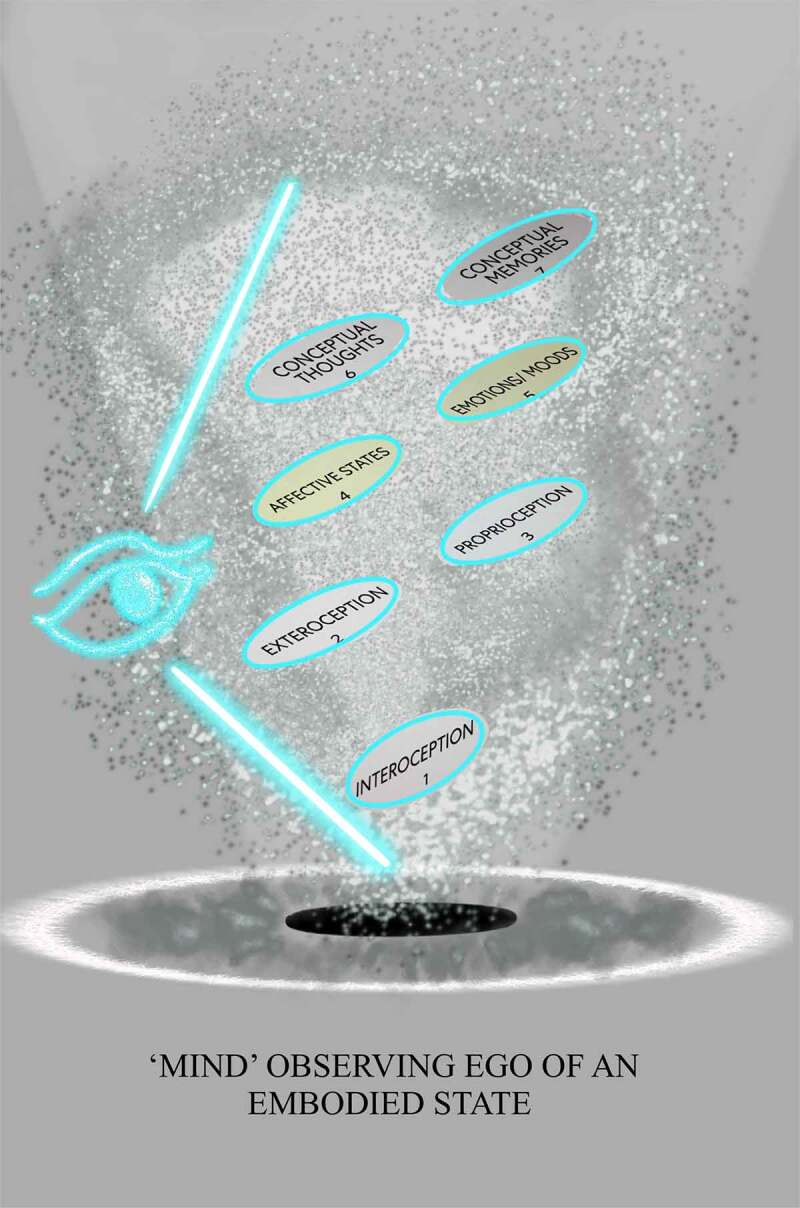
‘Mind’ observing ego of an embodied state.

‘Mind’ observing egos are emergent properties through embodied states with a mind dark energy substrate. A ‘mind’ observing ego represents an individual ”eye of the mind” or ”elusive self.” It is a specialized interoceptive awareness and magnetic anomaly on a cyclic, although not repetitive journey, interoceptive cognition consciousness. Through cognitive broadcasting, an observing ego creates a world that it projects around itself. A process understood through the components map model with interoceptive markers (IMs).

**Figure 16.**
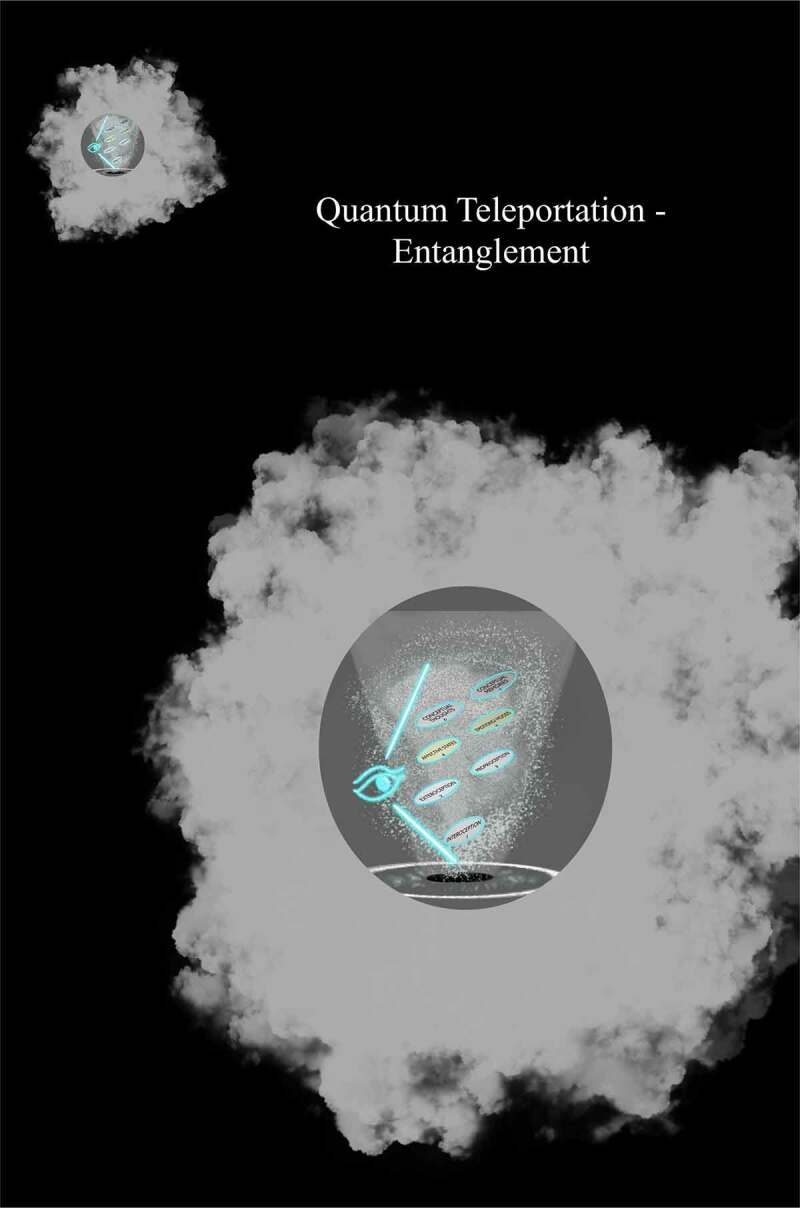
Quantum teleportation – Entanglement.

Current science does not support the notion of faster-than-light communication. However, in this theory, faster than light signaling is possible through mind dark energy representing pure awareness intelligence and unpatterned change (also specialized homogeneous change) that is not entirely conceivable. According to this theory, quantum entanglement is a phenomenon where instantaneous changes transmit across any space distance through mind dark energy. Signals faster than light speed travel on the pure awareness hidden level that is a substrate for all embodied states.

**Figure 17.**
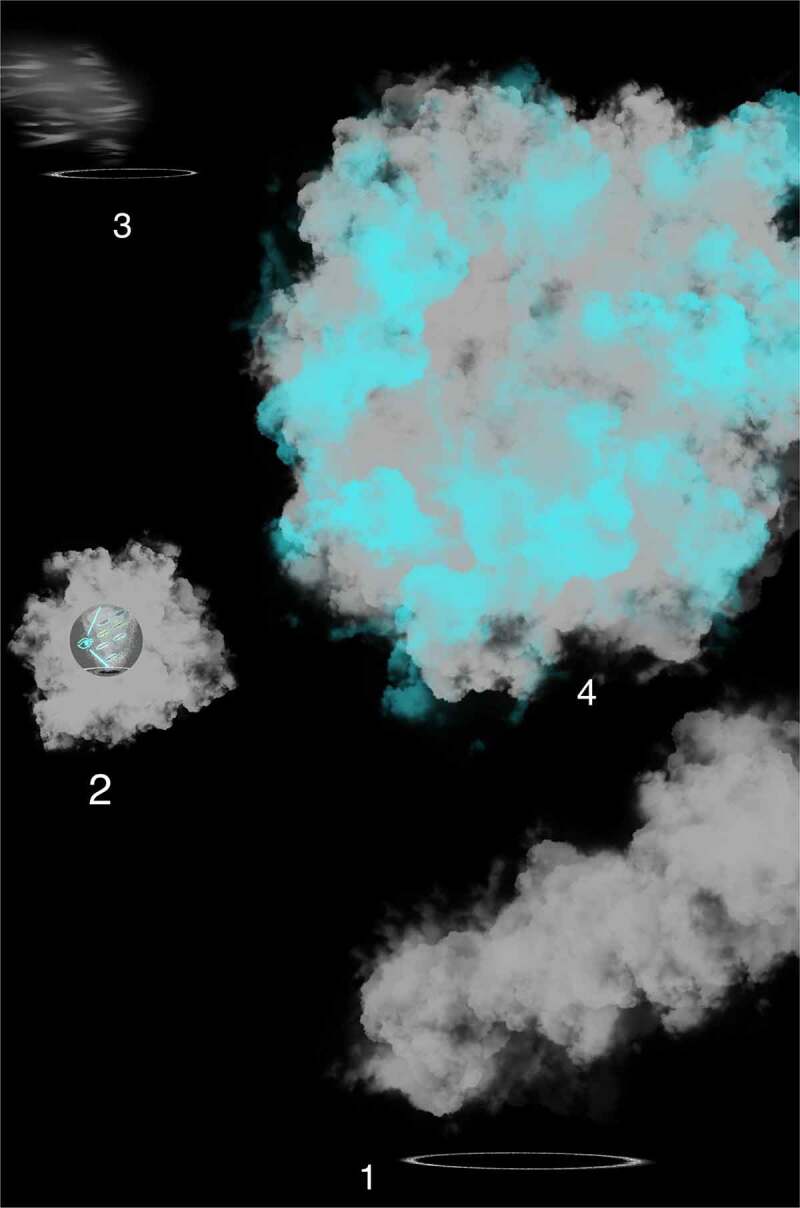
Four periods of cyclic existence.

This FoK-FIP theory builds upon Buddhist text in which there are four periods of cyclic existence: formation, continuity, destruction/nonmanifest state, and enlightened state:(1)Formation: The period where mind dark energy morphs with open and closed strings into mind FPDMs and includes their being embodied by mind normal matter. (2) Continuity: The period in which mind FPDMs embodied interacts with mind normal matter. During this period emerges 'mind' observing ego that cognitively broadcasts. (3) Destruction/Nonmanifest state: Through embodied states, this period is triggered by the inner layer of strings of mind FPDMs in which signals normal matter to process for impermanence. This period ends when mind a FPDM is no longer embodied by mind normal matter. As such, the nonmanifest state is a period that contains an intermediate void of the interdependent relationship between mind FPDMs and mind normal matter through an embodied state. (4) Enlightened: This image depicts an ”enlightened” embodied state where an observing ego is implied that can anticipate FoK-FIP. It decides to commit to a particular volition reaction as goal-directed action to benefit ”others” in this Universe. As such, enlightenment is a term used here to describe a 'mind' observing ego that has courageously changed its relationship to FoK-FIP. Due to subjective processes, this observing ego became aware of itself as the invisible viewpoint while perceiving its close relationship to a living organism model. Because cognitively broadcasting differently, the living organism model became the visual indicator that provided feedback to this observing ego of its ability to anchor its reactivity to FoK-FIP ultimately. While cognitively broadcasting, this 'mind' observing ego has willfully initiated volitional reactions that have irradicated its ignorance with an abundance of subjective processing that includes empathy with an absence of apathy. An observing ego has significantly impacted how it perceives the world it creates and projects around itself in this process. Through skilled cognitive broadcasting, this 'mind' observing ego can create the change it wishes to see in the physical world. Through its relationship to mind a FPDM, this change can spread through the universal network facilitated by mind dark energy to benefit “others” while they struggle to anchor their reactivity to FoK-FIP as the players of complex ”mind games.”

### FoK-FIP, selfish genes vs. the selfish open string, and the unselfish closed string

Perhaps, a new way of looking at humans may be one of the most defining concepts of the Selfish Gene theory (see Dawkins, 1976) [60]. Behavior results from the selfish gene, but not the selfish individual. In this highly rational and mathematical theory, the immortal soul is reinvented as a computer code that makes no distinction between good and evil. Instead, the DNA is a coded description of ancient worlds and wisdom out of the past. According to evolutionary biologist Richard Dawkins, we are simply machines playing minor roles in a vast strategic game played by competing computer codes over centuries. As such, DNA is analogous to the binary digits of a computer code, unreeling like a reel of magnetic tape on a giant computer. The Selfish Gene theory builds upon concepts derived from other theories including those of William Donald Hamilton FRS, a British evolutionary biologist, and evolutionary theorists of the 20th century whose theories implied that natural selection brought to its extreme conclusion is logical [61,62]. According to this view, nothing should be allowed to interfere with the strategy of the genes because some people are genetically inferior to others. Implied in this view is that it is wrong to use Western science and medicine to prolong the lives of those who would otherwise die because it would allow the genetically inferior to survive and, therefore, weaken society. This view suggests that only through modern medicine and social policies are these people kept alive, and the human species, because of this, will degenerate. According to this view, it is logical to show a desire to harm or defeat someone, as shown through formulas in which murder, warfare, and even genocide in a mathematical sense can be rational. In contrast, the unifying theory upon which this FoK-FIP theory is based (see Pollard-Wright, 2021) challenges the position that murder, warfare, and genocide are rational. Additionally, natural selection brought to its extreme conclusion is illogical when viewed with the established set of attitudes held by the mind that through ‘mind’ observing egos becomes cognitively aware of itself. It is logical to benefit “others” because there are no ”others,” fundamentally just the same kind of difference. Through a ‘mind’ observing ego, the mind can create a mindset that can be summarized here as ‘Eye/I’ stands for ‘you.’ According to this transdisciplinary theory, the concept of genetically inferior is an illusion that occurs through a ‘mind’ observing ego that cognitively broadcasts with more or less FoK-FIP sensitivity. In this transdisciplinary theory, the selfish gene is replaced by the selfish open string and its counterpart the unselfish closed string.

This Universe is posited to be a connected information system because of mind dark energy’s pure awareness state. Further, mind FPDMs each with its position on the Universe’s substrate mind dark energy, act as “cosmic brains” with cosmic-like instantaneous synapses and synapses with delay. Unpatterned data travels through the pure awareness state of mind dark energy straight over to the circles of compacted multi-dimensional space of mind FPDMs. In this process, the circle of dark energy (i.e., compacted multi-dimensional space) of mind FPDMs act as instantaneous cosmic synapses related to static magnetic fields. At the same time, the inner and outer layers of dark energy strings of mind FPDMs act like cosmic synapses where delay occurs between charged particles (i.e., closed and open strings). Each mind, a FPDM has the unique capacity through its inner and outer layer of open and closed strings to decode and store information while propagating new information based on the information stored. Through mind dark energy, unpatterned information is received by mind FPDMs in which the oscillations of the inner and outer layer of open and closed strings act as cosmic synapses. Through mind FPDMs, occurs bottom-up encoded signaling analogous to the binary digits of cosmic computer code. The oscillations of some closed strings on the inner layer of mind FPDMs cause the emergence of ‘mind’ observing egos, magnetic anomalies of embodied states.’ Mind’ observing egos define EMR consciousness, giving information contained with EM waves meaning. This cognitive broadcasting process produces meaning, resulting in interoceptive cognition consciousness. The reactions of ‘mind’ observing egos while cognitive broadcasting is how decisions are made. The volitional reactions of ‘mind’ observing egos cause the conversion of mind FPDMs strings (i.e., open strings convert to closed strings or closed strings convert to open strings). Current science does not support the notion of faster-than-light communication. However, this transdisciplinary theory makes faster-than-light signaling possible through the pure awareness state associated with mind dark energy. Through the unpatterned intelligence of mind dark energy that cannot be calculated or estimated is how facilitated faster-than-light communication between embodied states occurs. As part of the Universal system, mind FPDMs function as the mechanism in which vital information unpatterned through mind dark energy converts to data across time through open and closed oscillating dark energy strings.

In this theory, quantum entanglement is when instantaneous changes transmit across any space distance between mind FPDMs through mind dark energy that is the substrate for all embodied states. Through the relationship that each mind a FPDM has with mind dark energy, quantum signals can be registered and decoded in which quantum entanglement with instantaneous changes occurs. As such, a physical phenomenon occurs when a group of FPDMs interacts or shares spatial proximity of mind dark energy so that the quantum state of each FPDM (through inner and outer oscillating string layers) cannot be described independently of the state of the others. However, the interdependent relationships through embodied states set the limits of the quantum entanglement process. Each mind a FPDM, has a relationship with mind normal matter and a ‘mind’ observing ego while receiving unpatterned information from mind dark energy. Through mind FPDMs, unpatterned information can be received and decoded into patterned data by the oscillations of open and closed strings. Nevertheless, the data contained in EM waves produced through oscillating strings cannot be converted into meaningful information faster than light through ‘mind’ observing egos that cognitively broadcast. In a manner not entirely conceivable (due to the substrate of mind dark energy with the pure awareness state), posited is that mind FPDMs can know each other through the quantum entanglement process. Further, the dark energy strings of mind FPDMs are carriers through temporary patterns of oscillations that pass on copies of themselves. Congruent with FPDMs that can know each other is ‘mind’ observing egos that can know each other cognitively and thus differ from the way mind FPDMs know each other. Importantly, through bottom-up encoded signals received from mind FPDMs connected to a Universal network, ‘mind’ observing egos broadcast cognitively. When ‘mind’ observing egos engage in the subjective cognitive broadcasting process, the world projected around them comes from data in the EM waves from which observing egos emerged. This process is closely related to a particular living organism model (to varying degrees). Each ‘mind’ observing ego, through higher-end cognition, has a relationship with a living organism model (also Avatar living being) and derives a conceptual sense of self that it will use to understand their embodied situation (to varying degrees).

This transdisciplinary theory includes a model of the Universe in which a rise of primordial consciousness through the matrix of mind FPDMs is possible due to ‘mind’ observing egos’ inability to anchor their reactivity to FoK-FIP ultimately. Feedback loops are how mind FPDMs act as a bottom-up input encoding system in which the volitional reaction of ‘mind’ observing egos feeds into and return to shape the objective processing of signals. An observing ego can become attached or aversive to pleasant or unpleasant FoK-FIP. The volitional reactions of an observing ego act as feedback to mind a FPDM that causes string conversion that either change the way the inner layer of strings centers physiological signals and sensations or feelings or remains status quo. In this process, bottom-up encoded signaling updates to more accurately reflect the varying grotesqueries of a ‘mind’ observing ego’s nature based on a history of volitional responses to FoK-FIP ultimately. The mental stability of a ‘mind’ observing ego is dependent on its relationship to FoK-FIP, in which subjective processing of signals changes or remains status quo as a function of volitional reactions. Nearly all ‘mind’ observing egos accept bottom-up signaling of mind FPDMs as long as they perceive they have a choice, even if they were only aware of the choice at a near unconscious level. FoK-FIP acts as a powerful driver of universal complexity. Through varying degrees of ‘mind’ observing egos’ sensitivity, FoK-FIP centers sensation or feelings that act as an antecedent to emotion or moods. Observing egos’ nature is based on their sensitivity to FoK-FIP reflected through the cognitive broadcasting process that creates meaning from signals. With varying sensitivity, posited is that observing egos broadcast for higher-end cognition using FoK-FIP in which illusions manifest. Through cognitive broadcasting, observing egos wrongly perceive or interpret by the senses signals that appear more stable than they genuinely are. Further, observing egos believe themselves to be free to make choices unaware of FoK-FIP’s role in this process. The ability of each ‘mind’ observing ego to take action through volitional reactions freely is constrained through their sensitivity to FoK-FIP, ultimately. Through observing egos’ relationship to mind FPDMs with converting strings, an unpredictable level of complexity is added to a Universe with primordial-stabilizing systems.

Cyclic existence occurs in this theory that includes ongoing relationships between mind a FPDM with converting strings and a ‘mind’ observing ego on a journey through interoceptive cognition consciousness in which freely choosing to go on the journey was not part of the process. Instead, their journey is about the mind having manifested its infinite potential, which caused the change that resulted in this Universe with three types of consciousness. Each embodied state is how the mind simultaneously plays complex “mind games” in which interoception is a very secure system protecting an embodied state through FoK-FIP. Every signaling event triggers the inner string layer in which FoK-FIP, through specialized self-generated biomagnetism, centers signals and sensations or feelings. However, interoception has a weakness like all systems: ‘mind’ observing egos representing the cognitive force that broadcasts using FoK-FIP, including higherend cognition. Envisioned through the component map model with IMs are bottom-up signals of mind FPDM, and thus objective processing is received and defined by an observing ego. Each numbered component represents an observing ego’s subjective processing that creates the meaning of the information contained in EM waves. A component is a subsystem within a more extensive interoceptive cognition consciousness system. One component can affect other subsystems through causal relationships, and a ‘ripple effect’ can spread through the whole system. If an observing ego willingly changes its relationship to FoK-FIP through goal-directed reactions, this converts strings of mind a FPDM and all objective processing connected. As such, each ‘mind’ observing ego is a failsafe of an embodied state that can willingly change its relationship to FoK-FIP by initiating volitional reactions that take it to the gateway level. The gateway way level is congruent with signaling that can lead to many places, hidden mental content, but one component, affective states (i.e., component 4), is special. This component connects to the source of FoK-FIP, interoception (i.e., component 1) that holds the key to a ‘mind’ observing ego’s mental stability. An observing ego is the one that can open the door to its mental stability, but only with a willingness to anchor its reactivity to FoK-FIP at the gateway can the door be opened ultimately. With mental stability and a relationship to a human model, an observing ego can more or less understand the rules of the complex mind game of which it is a player.

Perhaps, a defining concept of this transdisciplinary theory is that no matter how much ‘mind’ observing egos may think they are free individuals, in reality, they are always running from mind FPDMs’ bottom-up encoded FoK-FIP. While unable to directly perceive themselves as invisible viewpoints or mind FPDMs, this relationship is known by ‘mind’ observing egos through FoKFIP that center their sensations or feelings. This process with the proposed mechanism” plays out” through higher-end interoceptive cognition in the world events mind observing egos create and project around themselves (to varying degrees). Accordingly, underneath the complexity of nature and the world’s ecosystems is a universe of cosmic-like ecosystems of embodied states. Our Universe is posited to consist of FPDMs that are the primordial-stabilizing part of embodied systems that can create an unpredictable order or a kind of unpredictable disorder while in relationship with ‘mind’ observing egos. This idea is congruent with all living organism models such as animal and plant models linked through ecosystems. What is not included in this theory is a belief in the balance of nature that can stabilize in a machine-like fantasy. Instead, behind every living organism model is an embodied state of mind in which this theory predicts very strange things about how matter works that are completely at odds with how things seem to work in the ”real world” that ‘mind’ observing egos project around themselves. The Universe and the three types of consciousness posited cannot be separated or excluded from each other. Instead, what occurs through embodied states gets reflected in the events of the ”real world” that each ‘mind’ observing ego creates and projects around itself. This process includes heating as the main biological effect of EMR consciousness that ”plays out” when ‘mind’ observing egos broadcast for higher-end cognition. Above the gateway level are conceptual thoughts (i.e., component 6) and conceptual memories (i.e., component 7) in which images emerge that include human models driven by attachment and aversion. The storyline associated with these models is that their impulsive reactivity causes insatiable activity resulting in global warming as increased levels of carbon dioxide, chlorofluorocarbons, and other pollutants are created to the detriment of other living organisms models (e.g., plants and nonhuman species of animals).

When ‘mind’ observing egos have “current concerns” for self above all “others” that are primarily nonconscious FoK-FIP driven, posited is that the mind cannot benefit itself. As such, the mind loses while playing its mind game through the goal-driven reactions of ‘mind’ observing egos. In contrast, when ‘mind’ observing egos overcome preference barriers that arise through sensing specialized small magnetic signals, and experience four phases of a narrowed range of interoceptive signals while having “current concerns” for others, the mind can benefit itself. As such, the mind wins while playing its mind game through the goal-driven reactions of ‘mind’ observing egos. Further, each “mind’ observing ego has a cyclic although not repetitive life that is the sum of an unbalanced equation inherent to its relationship with mind a FPDM with converting strings. Each mind a FPDM with converting strings acts as the ”banker” and data storage process that mirrors changes occurring through embodied states of mind. The conversion of strings acts as the record of oscillatory motion left by the current volitional reactions of a ‘mind’ observing ego and what they have been. Higher-end cognition displays “the mind game” through conceptual thought or conceptual memory in which associations between living organisms (Avatar living beings) occur. Through five symbiotic relationships that classify the game’s dynamics, understood is that the mind becomes everything to itself, through the perceptions of ‘mind’ observing egos as the players of the complex mind game:

1. Mutualism is a relationship between Avatars of two or more species that both benefit from the association as a net benefit.
2. Commensalism: is a relationship between Avatars of two species in which one benefits from the association (e.g., obtains food) without harming the other.
3. Predation: a relationship in which one Avatar (i.e., predator) kills and eats another Avatar (i.e., prey).
4. Parasitism is a relationship between two different Avatars whereby one (as the parasite) receives benefits from the other Avatar (the host), and the parasite harms the host.
5. Competition: is a relationship between Avatars with a rivalry or contest for resources, recognition, group, or social status.

## Conclusion

It has been proposed that academics are working on interoception without necessarily sharing the same conceptual base or necessarily realizing how their investigations link with the findings and insights of others [35]. The hypotheses and research proposed in this article were formulated using multidisciplinary and cross-disciplinary perspectives. As such, the approach includes the perspective of several related disciplines (e.g., psychology, neuroscience, neurology, psychiatry, ecology, psychophysiology, and physics) with the intent to further the study of interoception across behavioral sciences. The research proposed in this article may help stimulate transdisciplinary research that goes toward eliminating conceptual and methodological challenges that have made it difficult for interoceptive constructs to be broadly applied in transdisciplinary research and treatment settings [15].
